# TAM-targeted nanomedicine in cancer: biological basis, therapeutic strategies, and translational perspectives

**DOI:** 10.7150/thno.132714

**Published:** 2026-06-17

**Authors:** Lili Zhou, Fang Yuan, Yangyang Wu, Xin Li, Debiao Xiang

**Affiliations:** 1 Hunan University of Chinese Medicine, 300 Xueshi Road, Yuelu District, Changsha, Hunan Province, 410208, China.; 2 Department of Pharmacy, The Third Hospital of Changsha, 176 Western Laodong Road, Tianxin District, Changsha, Hunan Province, 410015, China.; 3 Hunan Provincial Key Laboratory of Anti-Resistance Microbial Drugs, Changsha, Hunan Province, China.; 4 Experimental Research Center, China Academy of Chinese Medical Sciences, Beijing 100700, China.; 5 Hunan University, No. 2, Lushan South Road, Yuelu District, Changsha, Hunan Province, 410082, China.

**Keywords:** tumor-associated macrophages, nanomedicine, macrophage reprogramming, biomimetic drug delivery, clinical translation

## Abstract

Tumor-associated macrophages (TAMs) are the most common immune cell type found in the tumor microenvironment. They are also key participants in the regulation of angiogenesis, metastasis, immune evasion, and therapeutic resistance. Recent single-cell transcriptomics and spatial profiling have shown that TAMs are extremely heterogeneous and functionally plastic beyond the traditional M1/M2 paradigm, and that more refined therapeutic strategies are required. Nanomedicine can integrate macrophage biology, the tumor microenvironment, and the engineering of the delivery system to design TAM-oriented interventions. With optimization of delivery systems (e.g. particle size, surface chemistry, ligands, therapeutic payloads, and the type of stimuli), multiple functionalities can be engineered to improve macrophage (in the tumor microenvironment) recruitment, depletion, reprogramming, and enhancement of TAM phagocytosis, antigen presentation, and immune regulation. Nanoparticles with biomimetic systems, macrophage membrane coating, as well as macrophage-derived extracellular vesicles and engineered exosome platforms, can also provide a wide array of options for TAM-oriented therapy. In this review, we provide an overview of the origin, heterogeneity, and functions of TAMs in cancer, and categorize TAM-targeted nanotherapeutics based on the principal approaches of recruitment blockade, tumor-promoting macrophage depletion, reprogramming, functional enhancement of phagocytosis and immune activation, and biomimetic drug delivery. We also present a comparison of representative nanoplatforms, highlight the latest clinical developments, and investigate key barriers to moving research from the lab to the clinic using nanomedicine, including delivery system design focused on targeting TAMs, safe and effective delivery to tumors, off-target effects on macrophages, ease of manufacture, and control of product quality. By combining TAM biology with the design of specialized nanocarriers and clinical research, this review outlines the main directions of research and provides a practical approach to the development of specialized TAM-targeted cancer nanomedicines.

## 1. Introduction

The tumor microenvironment (TME) is a rapidly evolving system consisting of malignant cells, tumor stroma, an extracellular matrix, and immune cells. Tumor associated macrophages (TAMs) are gaining recognition as crucial components of tumor biology, immune system refashioning, and treatment response, and represent viable therapeutic targets 1-3. The development of new technologies including single cell sequencing, spatial transcriptomics, and advanced immune profiling have helped researchers study TAMs and provide a more comprehensive analysis of their biology. These studies show that TAMs should not be studied by the traditional M1/M2 macrophage model, but their heterogeneity and plasticity should be taken into account 4-6. In many solid tumors, TAMs are mainly of monocytic origin, although in some cases, they are of macrophage origin and are influenced by tumor and tumor stroma derived factors like CCL2 and CSF1. Tissue-resident macrophages may also be influenced, but it is largely dictated by the tumor’s anatomical and biological environment 7-9. In the TME, TAMs can assume numerous phenotypically plastic states by local and intercellular factors. In many tumor types, including gastric cancer, lung cancer, and osteosarcoma, aggressive tumor stroma is associated with high density of macrophages and an unfavorable prognosis 10,11.

In a variety of ways, tumor-associated macrophages (TAMs) facilitate tumorigenesis. TAMs release vascular endothelial growth factor (VEGF) and matrix metalloproteinases (MMPs), which assist in angiogenesis and in the remodeling of the extracellular matrix (ECM). MMPs aid lymphangiogenesis and metastatic spread through the VEGF-C/VEGFR-3 pathway. TAMs also release immunoregulatory cytokines and express immune checkpoints, which aids the resistance to tumor immunity, and activate the PI3K/Akt pathway and STAT3, which facilitate the resistance to therapies 12-15. TAMs are involved in remodeling the stroma, and aid the tumor in adapting its metabolism and aid remodeling of the immune system following therapy. The centrality of TAMs in the remodeling of the microenvironment in tumors gives substantial support for developing therapies that deplete, reprogram, and functionally redirect TAMs.

Several TAM-directed therapies, including inhibition of CSF-1R and blockage of chemokines, have shown promise. A lack of sufficient specificity, systemic toxicity, and compensatory immunosuppressive mechanisms, as well as the context of the tumor, limit their use in the clinic 16. In this scenario, nanomedicine has sparked a lot of interest, as it is an adaptable and novel way for precision targeting of TAM modulation. Nanocarriers can improve drug targeting, provide controlled release, and support preferential uptake by macrophage subsets through tunable and stimulus-responsive properties 17,18. At the same time, accumulating evidence indicates that the success of TAM-oriented nanomedicine depends not only on payload selection, but also on how carrier size, surface chemistry, ligand presentation, penetration capacity, and manufacturability are matched to macrophage biology and intratumoral distribution 19-21.

In this review, we provide a systematic overview of the biological basis and therapeutic targeting of TAMs in cancer, with particular emphasis on nanomedicine-based strategies. We first summarize the ontogeny, heterogeneity, and functional states of TAMs, and then discuss their roles in angiogenesis, lymphangiogenesis, metastasis, immune evasion, and therapy resistance. We subsequently organize TAM-targeted therapeutic strategies into major mechanistic categories, including blockade of macrophage recruitment, depletion of tumor-promoting macrophages, reprogramming of macrophage polarization and function, restoration of phagocytic and immune-activating capacities, and exploitation of biomimetic or macrophage-derived delivery platforms. Finally, we discuss current clinical candidates, major translational barriers—including specificity, penetration, safety, and manufacturability—and future perspectives for the rational development of next-generation TAM-targeted nanotherapeutics. By integrating advances in TAM biology, nanocarrier engineering, and translational oncology, this review aims not only to summarize current TAM-targeted nanoplatforms, but also to clarify how macrophage heterogeneity and tumor context should guide the rational design of clinically translatable nanomedicine strategies.

## 2. The origin, plasticity, and heterogeneity of TAMs

### 2.1 Cellular origins of TAMs

Macrophages are a heterogeneous population of innate immune cells that play essential roles in host defense, antigen presentation, and tissue homeostasis 22,23. Under physiological conditions, tissue macrophages may arise either from embryonically derived tissue-resident progenitors or from circulating bone marrow-derived monocytes that infiltrate tissues and subsequently differentiate into macrophages. As indicated in studies 24,25, due to varying developments, macrophage populations across tissue exhibit context-dependent heterogeneity.

In tumors, tissue macrophages and blood monocytes recruited to tumors can contribute to the formation of tumor-associated macrophages (TAMs) 26,27. In the tumor microenvironment (TME), the accumulation of TAMs is influenced by the presence of certain growth factors and specific chemokines, and is especially characterized by the CCL2/CCR2 and CSF-1/CSF-1R signaling pathways 28,29. As one of the most abundant immune cell populations in the TME, TAMs are continuously shaped by soluble mediators released from tumor and stromal cells, as well as by interactions with other immune cells. This ontogenetic diversity is therapeutically important, because recruitment-blocking strategies may preferentially affect monocyte-derived TAM populations rather than all macrophage subsets 30.

### 2.2 Functional polarization and plasticity of TAMs

**Figure 1:**
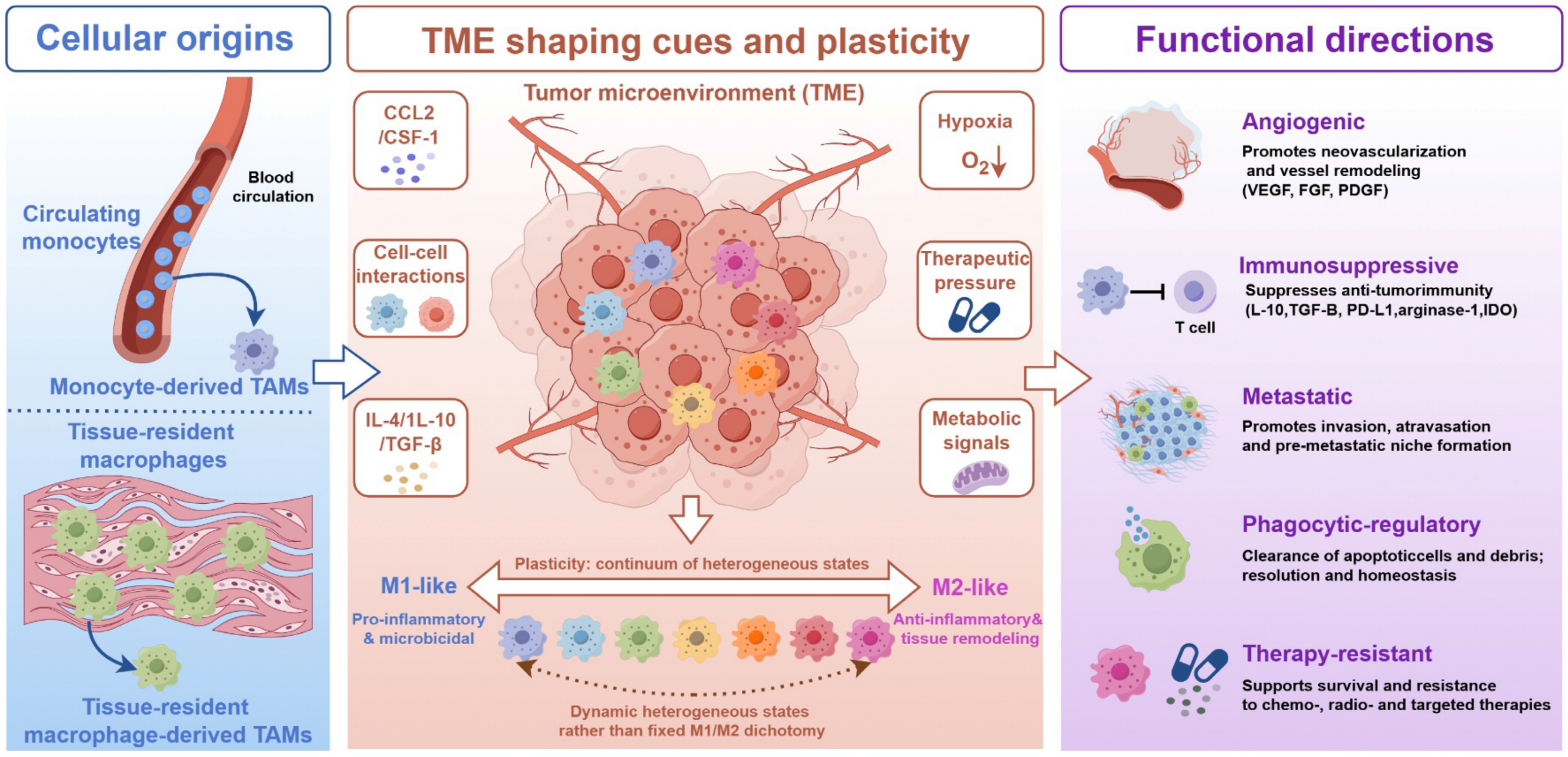
** Cellular origins, TME-driven plasticity, and functional heterogeneity of TAMs.** Tumor-associated macrophages (TAMs) arise from both circulating monocytes and tissue-resident macrophage populations. Circulating monocytes can be recruited into tumors and differentiate into monocyte-derived TAMs, whereas tissue-resident macrophages may adapt to local tumor cues and give rise to tissue-resident macrophage-derived TAMs. Within the tumor microenvironment (TME), TAMs are continuously shaped by soluble mediators and functional heterogeneity of TAMs. Tumor-associated macrophages (TAMs) arise from both circulating monocytes and tissue-resident macrophage populations. Circulating monocytes can be recruited into tumors and differentiate and local stress signals, including CCL2/CSF-1, IL-4, IL-10, TGF-β, hypoxia, metabolic signals, therapeutic pressure, and cell–cell interactions. These cues drive macrophage plasticity and generate a continuum of heterogeneous TAM states rather than fixed M1/M2 categories. M1-like and M2-like phenotypes are shown as functional reference states, ranging from pro-inflammatory and microbicidal programs to anti-inflammatory and tissue-remodeling programs. Functionally, TAMs can contribute to angiogenesis, immunosuppression, metastasis, phagocytic regulation, and therapy resistance, thereby providing the biological basis for TAM-targeted nanomedicine strategies.

Macrophages exhibit remarkable functional plasticity and dynamically respond to microenvironmental cues 31. Although the M1/M2 framework remains useful for describing opposite functional tendencies, it does not fully capture the diversity of TAM states observed in tumors 32. Classically activated M1-like macrophages generally occupy the pro-inflammatory end of the spectrum, whereas alternatively activated M2-like macrophages are more commonly associated with anti-inflammatory, tissue-remodeling, and immunoregulatory functions 33. Within the TME, factors such as hypoxia and tumor-derived suppressive signals frequently skew macrophages toward immunosuppressive, tumor-supportive states 34. This plasticity provides an important biological rationale for therapeutic strategies aimed at reprogramming TAM function. Resting macrophages can undergo functional polarization in response to local microenvironmental stimuli, giving rise to a range of activation states that are often described using the M1/M2 framework (Figure 1) 35. Importantly, the M1-like and M2-like states are used here as functional reference states rather than strict classifications of intratumoral macrophage populations 36.

M1-like macrophages are typically induced by IFN-γ, LPS, and other Th1-associated signals. They commonly present increased inflammatory markers, including MHC-II, TLR4, CD80, and CD86, alongside improved antigen-presenting ability 37. These macrophages can synthesize the pro-inflammatory cytokines IL-1β, IL-6, and IL-12, and TNF-α, and can have anti-tumor effects through the release of ROS and NO, which can frequently be associated with the upregulation of iNOS. All of these provide significant justification for the proposed immune-supportive repolarization of TAMs in this context. However, as suggested by the name, too much or prolonged inflammatory activation may result in damage to other tissues and cause chronic inflammation. It can be considered that repolarization of TAMs in this manner, in most cases, should be a controlled process rather than a free and unruly one.

On the other hand, M2-like macrophages are typically identified with anti-inflammatory, pro-angiogenic, and tissue remodeling functions. In tumors, they tend to promote immune evasion, matrix remodeling, angiogenesis, invasion, and metastasis 38. They are even further characterized by an upregulation of Arg-1, CD163, and CD206. They are targeted, and functionally utilized, in TAMs mobilized nanomedicine.

### 2.3 Beyond the M1/M2 paradigm: heterogeneity of TAMs

While valuable historically and descriptively, the M1/M2 model captures a simplified version of TAM biology 39. In solid tumors, macrophages seldom exist as two discrete and stable populations. Instead, they occupy a dynamic and multidimensional spectrum of activation states, influenced by the type of tumor, stage of disease, the anatomical niche, metabolic conditions, stroma, and the use of various therapies 40,41. New technologies in single-cell transcriptomics and spatial profiling have even further underscored this complexity, identifying TAM subsets characterized by angiogenesis, matrix remodeling, immunosuppression, phagocytosis and therapy resistance and regulation 42, 43. This new perspective is particularly important for the design of nanomedicine. The targeting ligands, therapeutic cargoes, and intervention windows may depend on which macrophage spatially restricted subsets and programs are most dominant in a given tumor context 44,45.

In the past, M2 macrophages, which are often referred to as macrophages that are alternatively activated, have been subclassified as M2a, M2b, M2c, and M2d according to activating signals as well as functions 46-50. In this review, the M2-like states will be retained as reference categories for readability, but this will not be used to classify the taxonomy of macrophage populations that reside in tumors. In this context, Table 1 presents a variety of TAM states and functions that are relevant to nanomedicine. It will also be made clear that the heterogeneous nature of TAMs cannot be captured by the M2a-M2d macrophage classification. It must be noted that macrophage states are not achieved in a final and irreversible form 51. In the TME, cues that reside locally can lead to transitions to a newly functionally distinct macrophage program. This highlights the dynamic and highly context-dependent nature of TAMs.

The origin, heterogeneity, and plasticity of TAMs contribute to the complexity and allure in targeting these cells for cancer-nanomedicine. For example, a better understanding of the biology of TAMs is required when designing possible strategies that would attempt to block the recruitment of TAMs, deplete tumor-supportive TAMs, or alter TAMs to an anti-tumor supportive role. Thus, future TAM-focused cancer-nanomedicine should be based on more critically defined and clinically applicable states of macrophages, and not on overly simplified and rigid categorical frameworks.

## 3. Biological functions of TAMs in tumor progression: implications for therapeutic targeting

Expanding on the origin, plasticity, and heterogeneity of TAMs, this section describes the main biological functions through which TAMs drive the progression of tumors. Promoting angiogenesis and lymphangiogenesis, facilitating tumor metastasis, supporting immune evasion, and promoting resistance to chemotherapy and other forms of cancer treatment are functions of TAMs that drive cancer progression and affect the treatment of the disease. TAMs and their main functions associated with therapeutic resistance and cancer progression are summarized in Table 2 along with their associated therapeutic resistance.

### 3.1 TAMs and tumor angiogenesis

Tumor-associated macrophages (TAMs) play a central role in tumor angiogenesis and multisystem vascular remodeling. Clinical and experimental studies show that dense TAMs are positively correlated with microvessel density in several solid tumors (e.g., colon and gastric cancer). These studies suggest that TAM accumulation and tumor vascularization are positively correlated 52,53. Macrophage depletion studies support these findings as they show that lacking TAMs compromises the formation of new blood vessels and impedes the growth of tumors 54.

In terms of mechanisms, TAMs primarily promote angiogenesis by producing a multitude of soluble, proangiogenic factors, and via the remodeling of extracellular matrix and perivascular scaffolding. TAMs release a variety of factors such as VEGF, PDGF, and TGF-β, which all promote the proliferation and migration of endothelial cells and the remodeling of blood vessels. Among these factors, VEGF-A has distinct significance as an angiogenic factor. In addition to its role in the activation of endothelial cells, VEGF-A also promotes the expansion of tumor vasculature and further recruitment of macrophages to the tumor microenvironment. In addition, TAMs provide support for the remodeling of the extracellular matrix by secreting matrix metalloproteinases, which release growth factors and facilitate the invasion of endothelial cells 55,56. TIE2-expressing TAMs are one of the specialized perivascular TAM subsets that support a proangiogenic role and are linked with treatment failure and tumor recurrence 57,58.

Hypoxia exhibits key angiogenic functions. It activates macrophage recruitment and tumor-supporting polarization and hypoxia mediates the factor secretions of pro-angiogenic mediators. It also causes tumor-supporting TAMs to produce CSF-1 and CCL2, factors for recruitment and vascular remodeling. TAMs produce the most help for angiogenesis. CSF-1 mediates the polarization and recruitment of macrophages 59,60.

The hypoxia-mediated TAM-aided angiogenesis axis represents a pathway of therapeutic resistance. It causes poor tumor architecture and aids the growth of tumors and worsens the delivery of therapeutics. TAMs play a role in all tumor angiogenesis. Here is a basis to aid in the development of TAM-targeted therapeutics. The simultaneous recruitment, polarization, and signaling blockade are TMAs better targets and aid in therapeutic resistance. They will also aid in providing a basis of anti-angiogenesis therapy. Modulation of TAM recruitment signaling blockade combined with vascular framework nanoplatforms would aid therapeutic resistance tremendously.

### 3.2 TAMs and tumor-induced lymphangiogenesis

TAMs also regulate lymphangiogenesis and lymphatic spread related to tumors 61. Current evidence indicates that TAMs promote lymphatic vessel formation mainly through the VEGF-C/VEGF-D–VEGFR-3 signaling axis, thereby stimulating lymphatic endothelial cell proliferation, migration, and permeability and facilitating lymph node metastasis 62-64. Clinically, this process is highly relevant because increased lymphangiogenesis is closely associated with nodal spread, metastatic progression, and poor prognosis. Tumor-derived signals within the microenvironment can further enhance VEGF-C production by TAMs and reinforce a reciprocal interaction between macrophage polarization and lymphatic remodeling. Thus, TAMs not only promote lymphatic vessel formation but may also be driven toward more tumor-supportive states by lymphangiogenic signaling, establishing a feed-forward loop that favors metastatic dissemination.

Beyond the canonical VEGF-C/VEGFR-3 pathway, additional mechanisms such as hypoxia-associated signaling, prostaglandin-related pathways, and podoplanin-associated interactions have also been implicated in TAM-mediated lymph angiogenesis 65-67. However, the relative importance of these noncanonical mechanisms appears to be context dependent and remains less well defined than that of VEGF-C-centered signaling.

Overall, TAMs are closely linked to lymphatic remodeling and nodal dissemination, supporting the development of TAM-targeted therapeutic approaches aimed at limiting lymphatic metastasis through disruption of VEGF-C-related signaling and reprogramming of tumor-supportive macrophage states. Because lymphatic remodeling is closely associated with nodal metastasis, recurrence, and poor prognosis, TAM-directed strategies that disrupt VEGF-C-related signaling or reprogram lymphangiogenic macrophage states may have particular value in metastasis-prone tumors.

### 3.3 TAMs and tumor metastasis

TAMs contribute to metastasis at multiple stages of tumor progression, including local invasion, epithelial–mesenchymal transition (EMT), premetastatic niche formation, and metastatic colonization. High infiltration of M2-like TAMs is frequently associated with increased tumor invasiveness and poor clinical outcome, supporting their important role in metastatic dissemination 68. One major mechanism involves extracellular matrix remodeling. TAMs secrete growth factors and proteases that degrade structural barriers, facilitate tumor cell migration, and promote invasion into surrounding tissues 69.

A second major mechanism is the induction of EMT. A crucial step in the process of tumor cell migration and metastasis is losing the cell–cell adhesion characteristic of epithelial cells and gaining characteristics of migration, invasion, anaplastic cells, and a stem-like phenotype. Clinically, the epithelial-to-mesenchymal transition (EMT) process is associated with a poor prognosis due to increased resistance to therapy and increased metastatic potential. Several TAM-derived signals including TGF-β1, TNF-α, IL-10, and inflammatory signaling, as well as CCL18, a chemokine, have been associated with triggering EMT in various tumors 70,71. With these signals, TAMs may contribute to mesenchymal transformation and the acquisition of an anaplastic cell phenotype, thus connecting the metastatic spread of tumors with the resistance of tumors to therapy.

In addition to initiating EMT in a primary tumor, TAMs may contribute to the formation of a premetastatic niche. TAMs, with their ability to secrete a variety of molecules and change the structure of remote tissues, may contribute to the creation of favorable conditions that allow the capture, persistence, and growth of tumor cells that have spread. This shows that TAMs do not limit their activity of promoting metastasis to a primary tumor 72,73.

Exosome-mediated communication provides another important layer of TAM-associated metastatic regulation. Tumor-derived exosomes can reprogram macrophages toward tumor-supportive phenotypes, whereas macrophage-associated exosomes can in turn enhance invasion, EMT, and metastatic behavior of tumor cells. This bidirectional communication highlights the dynamic role of TAMs in metastatic progression and suggests that intercellular signaling through extracellular vesicles may represent an important therapeutic vulnerability 74,75.

Overall, multiple TAM-derived mediators have been implicated in EMT and metastatic dissemination, but their relative importance likely differs across tumor types and disease stages. These findings support macrophage reprogramming, anti-metastatic combination nanotherapy, and selected biomimetic delivery platforms as the most relevant strategy classes for interfering with TAM-driven metastatic progression. Because EMT is associated with metastasis, therapeutic resistance, and poor prognosis, TAM-targeted nanomedicine may be most useful when combined with anti-metastatic or chemotherapy-sensitizing strategies.

### 3.4 TAMs and tumor immune evasion

Immune evasion is a defining hallmark of tumor progression, and TAMs contribute to this process by suppressing effector immunity and reinforcing immunosuppressive networks within the tumor microenvironment 76. One key mechanism involves the inhibition of cytotoxic T-cell activity. TAMs can physically and functionally limit CD8+ T-cell infiltration into tumor nests, thereby weakening one of the major arms of antitumor immunity. Experimental blockade of macrophage recruitment, such as inhibition of CSF-1R signaling, has been shown to restore intratumoral CD8+ T-cell accumulation and improve the efficacy of immune checkpoint blockade, illustrating the therapeutic relevance of macrophage-dependent T-cell exclusion 77.

TAMs also contribute to the immune microenvironment using cytokine-mediated suppression. The combination of reduced IL-12 and an increase of IL-10 and prostaglandin E2 create a more suppressive microenvironment, lead to a loss of function of effector T cells, and recruit and produce more regulatory T cells. The presence of CCL17, CCL18, and CCL22 enhances the recruitment of Tregs and adds a more suppressive environment. TAMs can also enhance the immune suppressive environment by recruiting myeloid-derived suppressor cells. 78,79.

TAMs also escape immune surveillance by using checkpoint mechanisms and using antiphagocytosis mechanisms. PD-L1 expression on TAMs, leads to the engagement of PD-1 by T cells and causes T cell response suppression and dysfunction by galectin-9/Tim-3 signaling. TAMs also regulate the major “don’t eat me” signaling pathway, CD47/SIRPα pathway, that inhibit macrophage phagocytosis of tumor cells. These examples illustrate that TAMs are not just passive players of immune suppression, they are key players of immune escape mechanisms for both adaptive and innate mechanisms.

Immune regulation involves an added dimension of metabolic and enzymatic suppression. TAMs express and/or secrete IDs, arginase, iNOS, CD39, and CD73, among other immunoregulatory molecules. These can modify the availability of different nutrients and signal transduction systems, which in turn inhibit the immune response to tumors. The cGAS cGAS-STING signaling pathway of TAMs has the potential to enhance type I interferon response but has the potential to exert compensatory immunosuppressive effects by upregulating PD-L1. It is a good example of the paradox of macrophage-centered immune signaling. It is important to avoid judging TAMs to be uniformly and/or absolutely suppressive 82,83.

TAM-mediated immune evasion must be considered holistically and in the context of the cellular, checkpoint, and metabolic suppression. This justified the design of therapeutic strategies targeting TAMs, which integrate macrophage reprogramming with checkpoint blockade, restoring phagocytosis, CD40-targeted immune activation, and other immune modulatory strategies.

### 3.5 TAMs and chemotherapy resistance

There is increasing evidence that tumor-associated macrophages (TAMs) are critical to resistance to chemotherapy and other forms of treatment. Their impact is primarily through impaired drug delivery, impaired stem cell drug delivery, and exosome-mediated communication 84.

Body of evidence suggests that macrophage, and in particular, tumor-associated macrophage, mediated chemotherapy resistance involves signal and cytokines. Mediators such as IL-6, IL-10, IL-8 CCL2 and tumor-associated signaling and inflammatory cascade, facilitate tumor cell survival by activating STAT3, PI3K/Akt, NF-ĸB pathways, and diminishing the treatment-associated apoptosis thus increasing the tumor cell drug resistance 85. A second mechanism is vascular remodeling. By promoting abnormal angiogenesis and low-perfusion vascular networks, TAMs impair intratumoral drug penetration and thereby diminish chemotherapy efficacy 86.

TAMs also contribute to resistance by supporting cancer stem cell (CSC)-associated phenotypes. Through pathways such as IL-6/STAT3, TAMs can enhance stem-like traits, while CSCs may in turn promote macrophage differentiation toward tumor-supportive states, reinforcing a resistant niche 87. In addition, TAM-derived exosomes carrying regulatory microRNAs and other cargos can promote survival signaling, inhibit apoptosis, and reduce responsiveness to chemotherapeutic agents in recipient tumor cells 88,89.

Taken together, TAM-associated chemoresistance is driven by integrated signaling, vascular, stemness-related, and exosome-mediated mechanisms. These observations suggest that TAM-targeted therapeutic strategies may overcome drug resistance not only by reprogramming macrophage phenotype, but also by improving drug delivery and disrupting resistance-supportive intercellular communication 90.

**Figure 2:**
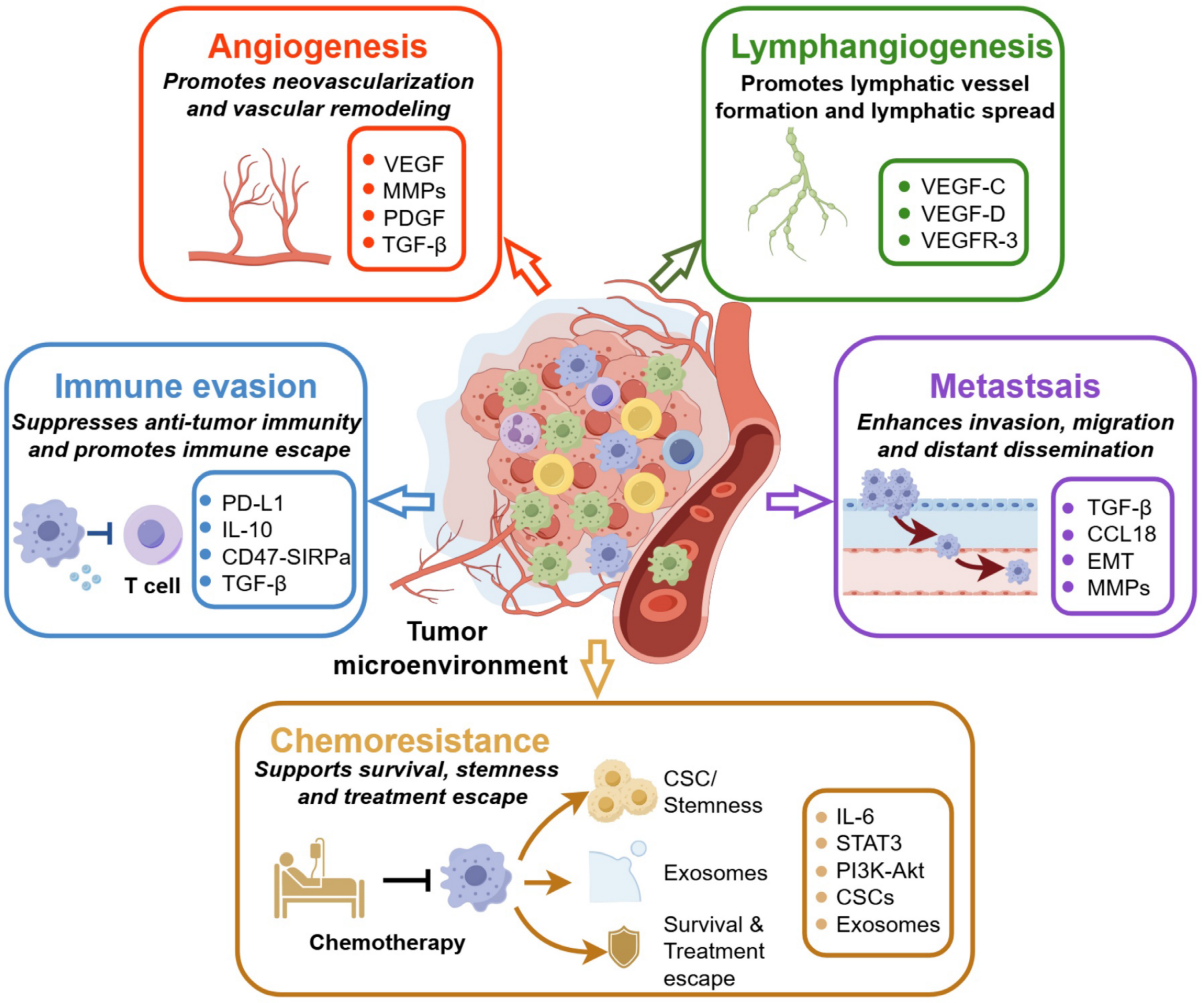
** Major biological functions of TAMs in tumor progression.** Tumor-associated macrophages (TAMs) promote tumor progression through multiple interconnected mechanisms within the tumor microenvironment. TAMs support angiogenesis by producing pro-angiogenic and matrix-remodeling mediators, including VEGF, MMPs, PDGF, and TGF-β, thereby promoting neovascularization and vascular remodeling. They also contribute to lymphangiogenesis through VEGF-C, VEGF-D, and VEGFR-3-related signaling, which facilitates lymphatic vessel formation and lymphatic dissemination. In addition, TAMs suppress antitumor immunity through immune-inhibitory mediators and checkpoint-related pathways, including PD-L1, IL-10, TGF-β, and CD47/SIRPα, resulting in impaired T-cell activity and immune escape. TAMs further enhance invasion, migration, and distant dissemination by promoting EMT and metastasis-associated pathways involving TGF-β, CCL18, and MMPs. Finally, TAMs contribute to chemoresistance by supporting cancer stem cell-like phenotypes, exosome-mediated intercellular communication, survival signaling, and treatment escape through pathways such as IL-6/STAT3 and PI3K/Akt. Together, these functions provide the biological rationale for TAM-targeted nanomedicine strategies aimed at blocking macrophage recruitment, depleting tumor-promoting TAMs, reprogramming macrophage function, restoring antitumor immunity, and improving therapeutic response.

Overall, TAMs influence tumor progression through multiple interconnected biological processes rather than through a single tumor-promoting mechanism. By regulating vascular remodeling, lymphatic spread, metastatic dissemination, immune suppression, and resistance to therapy, TAMs occupy a central position in the tumor microenvironment. Together, Table 2 and Figure 2 summarize these major TAM-associated functions and illustrate why they provide both the biological basis and the therapeutic rationale for the mechanistically organized strategies discussed in next section.

## 4. Anti-tumor nanomedicine strategies targeting TAMs

**Figure 3:**
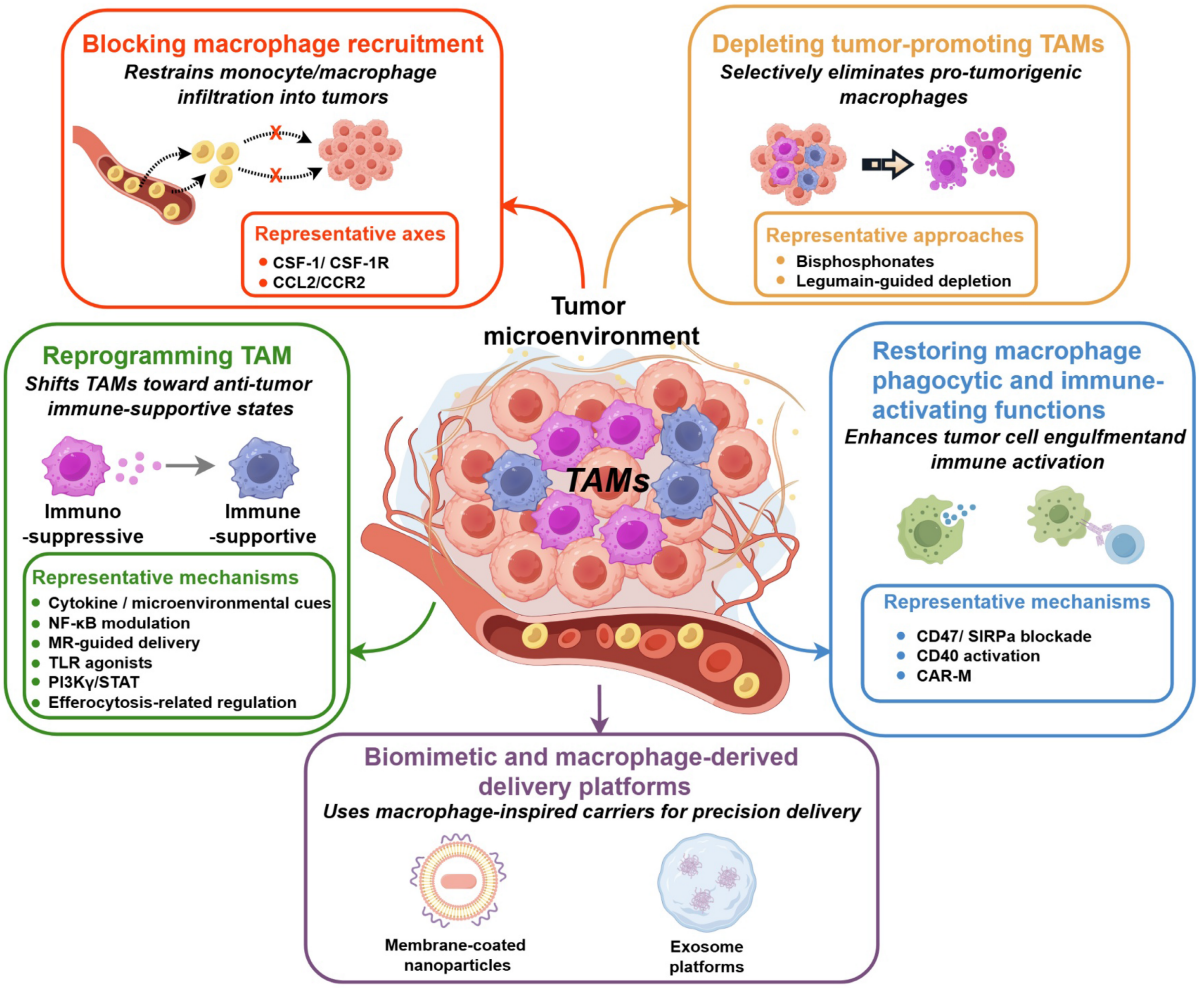
** Mechanistically organized TAM-targeted nanomedicine strategies.** Tumor-associated macrophage (TAM)-targeted nanomedicine can be organized into five major strategy classes according to the principal mode of macrophage intervention. First, blocking macrophage recruitment aims to restrain monocyte/macrophage infiltration into tumors, mainly through representative recruitment axes such as CSF-1/CSF-1R and CCL2/CCR2. Second, depletion strategies are designed to reduce tumor-promoting TAM populations, using approaches such as bisphosphonate-based macrophage depletion and legumain-guided depletion. Third, TAM reprogramming seeks to shift immunosuppressive macrophage states toward more immune-supportive phenotypes through cytokine- or microenvironmental cue modulation, NF-κB regulation, mannose receptor-guided delivery, TLR agonists, PI3Kγ/STAT signaling modulation, and efferocytosis-related regulation. Fourth, restoration of macrophage phagocytic and immune-activating functions focuses on enhancing tumor-cell engulfment, antigen presentation, and innate–adaptive immune activation through mechanisms such as CD47/SIRPα blockade, CD40 activation, and CAR-M-based strategies. Fifth, biomimetic and macrophage-derived delivery platforms, including membrane-coated nanoparticles and exosome platforms, serve as macrophage-inspired carriers to improve inflammatory homing, payload protection, and precision delivery. Collectively, these strategies demonstrate how nanomedicine can influence TAM recruitment, survival, functional state, and activity as well as delivery in the tumor microenvironment.

To provide an overview of the mechanistically organized TAM-targeted nanomedicine strategies discussed in this section, the major intervention classes and their associated engineering logic are summarized in Figure 3.

### 4.1 Blocking macrophage recruitment

#### 4.1.1 Targeting the CSF-1/CSF-1R axis

The colony-stimulating factor 1 (CSF-1)/CSF-1 receptor (CSF-1R) axis is a central regulator of macrophage survival, differentiation, and maintenance, and has therefore become one of the most intensively studied targets for limiting tumor-promoting TAM accumulation. In tumors, activation of this pathway promotes monocyte-to-macrophage differentiation, sustains TAM survival, and supports the establishment of a macrophage-rich microenvironment that facilitates tumor growth, angiogenesis, invasion, and metastasis. Accordingly, pharmacological inhibition of the CSF-1/CSF-1R axis has been widely explored as a strategy to reduce TAM abundance and remodel the tumor microenvironment 29.

Preclinical studies have demonstrated that CSF-1/CSF-1R inhibition can suppress tumor progression in multiple tumor models, including breast cancer and glioblastoma. Representative small-molecule inhibitors such as GW2580 and BLZ945 have shown the ability to reduce M2-like TAM infiltration, improve intratumoral immune conditions, and inhibit tumor growth 91,92. To address the challenges with free inhibitors, including their hydrophobicity, poor tumor selectivity, and increased systemic exposure, several nanoplatforms target tumor-associated macrophils (TAM) with greater delivery efficiency. For instance, DH@ECm, a pH-responsive micelle, improves TAMs’ BLZ945 uptake via CD206, while other BLZ-945SCNs/Pt combination nanocarriers integrate TAM removal with TAM-depleting chemotherapy for CD206-assisted chemotherapeutic delivery 93,94.

The design of these nanoplatforms at the microscale and macroscale directly impacts their product performance. For example, several criteria must be considered when determining the overall design of a nanoplatform meant to target TAMs and also enhance drug delivery to the tumor. This includes the size of the nanocarrier and its surface additives. A balance is required when optimizing these dimensions, as they may TAMs and drug conjugates in a tumor, but suboptimal dimensions will fail to reach TAMs in the tumor. Passive targeting may also be improved with surface design. Hybrid biomimetic membranes may promote passive targeting, while drug conjugates with pH-responsive surfaces may tune targeting to the tumor microenvironment. The nature of TAMs may also dictate which surface designs confer the best functional targeting to a TAM population. For instance, delivery to TAMs of the M2 phenotype may be improved with surfaces containing elements of sialic acid, M2pep, or CD206 targeting dextran. Thus, CSF-1/CSF-1R-directed nanomedicine with TAM targeting is enhanced through superior design of the nanocarrier to address TAM uptake and TAM phenotypes.

RNA-based approaches provide an alternative means of targeting this pathway with high mechanistic specificity. Nanoparticle-mediated delivery of CSF-1R-directed siRNA has been used to selectively suppress signaling in M2-like TAMs and reduce tumor-promoting macrophage populations. Representative examples include dual-targeting nanoparticles such as M2NP-siCD115, which combine SR-B1-targeting and M2pep-mediated recognition, as well as sialic acid-targeted cyclodextrin nanoparticles designed to enhance selective delivery of CSF-1R siRNA into M2 macrophages 95,96. In addition, combinational nanodelivery systems co-targeting CSF-1R and other immunoregulatory pathways, such as PI3Kγ, have shown stronger TAM-remodeling effects than single-pathway inhibition, including reduction of M2-like TAMs, increase of M1-like macrophages, and decreased infiltration of myeloid-derived suppressor cells. These findings suggest that CSF-1/CSF-1R blockade may be most effective when incorporated into multifunctional or combination-oriented nanomedicine designs rather than used as a stand-alone intervention.

The development of several CSF-1/CSF-1R-targeted agents, including the mAbs emactuzumab, AMG820, and IMC-CS4, is ongoing, with early-phase clinical trials underway, highlighting the translational impact of this pathway 97. However, adaptive resistance remains a pressing challenge. In glioblastoma models, compensatory paracrine signaling associated with the IL-4-mediated upregulation of IGF-1 has been shown to promote the activation of IGF-1R/PI3K signaling in tumor cells, allowing for the continued progression of the tumor, despite the blockade of CSF-1R 8. In the context of anti-PD-1 resistant liver cancer, increased expression of CSF-1 has been shown to drive the transformation of tumor-associated macrophages (TAMs) to a giant cell phenotype, which drives immune resistance and treatment failure. Overall, CSF-1/CSF-1R-targeted nanomedicine offers a good biologically based strategy to target tumor-associated macrophages (TAMs), especially in the tumors with high CSF-1 signals and macrophage density. However, adaptive resistance, suboptimal selectivity, and the need for a rational design of combinatorial approaches pose challenges to achieving extended clinical application. Hence, future improvements should incorporate a more biologically based approach to fine-tune TAMs in the tumor microenvironment through alterations in particle size, surface characteristics, and ligand presentation, rather than solely pathway targeting.

#### 4.1.2 Targeting the CCL2/CCR2 axis

Inflammatory monocyte recruitment is regulated by the CCL2/CCR2 axis and presents a novel approach to target tumor-associated macrophages (TAMs). In the tumor microenvironment (TME), CCL2 produced by tumor and stromal cells aids the recruitment of monocytes expressing CCR2 resulting in TAM infiltration, macrophage polarization, and cancer-related inflammation. This axis can be targeted to prevent an influx of myeloid cells to the tumor and may promote CD8+ T cell activity to inhibit tumor development 98.

At several levels including nanomedicine, tactics targeting the CCL2/CCR2 axis employ RNA interference, mRNA delivery, and design of receptor-targeted carriers. TAM abundance in models of lymphoma and colon cancer has been diminished through the use of lipid nanoparticles containing CCR2-targeted siRNA, indicating the potential of this approach to inhibit RNA-based monocyte recruitment 99. MC3 lipid nanoparticles with targeted liver delivery and bispecific CCL2/5i mRNA also demonstrated a decreased macrophage infiltration to the tumor by blocking both the CCL2 and CCL5 signaling pathways. 100. These approaches show that targeting multiple (or redundant) chemokine pathways may be more effective than targeting a single ligand in blocking recruitment within the TME.

Beyond pathway blockade, some nanoplatforms have also been developed to utilize CCR2-related biology to implement targeted drug delivery. An example is KLAK-MCP-1 micelles, which contain a motif that targets CCR2 and a pro-apoptotic peptide. This allows for the recognition of the CCR2 receptor and the delivery of cytotoxic payloads to tumor cells 101. Physicochemical design of these systems is crucial to their function. Micelles of smaller dimensions may favor tumor penetration. Additionally, the composition of the lipid nanoparticles may alter circulation, payload stability, and selectivity to certain organs. The selection of ligands also requires consideration of TAM biology. For example, targeting CCR2 or MCP-1 may favor delivery to inflammatory monocytes or tumor-associated CCR2-positive cells. However, these systems are not consistently selective. Therefore, the effectiveness of CCL2/CCR2-targeted nanomedicine not only requires blocking recruitment signals, but also needs to consider the design of the carrier and the targeted CCR2 positive cells 102.

Several CCR2-targeted agents like CCX872-B, BMS813160, PF-04136309, and MLN1202 have shown preclinical efficacy and some have entered clinical trials. These studies show that strong inflammatory monocyte recruitment to tumors may depend on the translation of this pathway 103. Yet crucial drawbacks were identified after conducting the first human trials. For CCL2-neutralizing antibodies, like carlumab, the preliminary evidence pointed to an antitumor effect but achieving durable suppression of the CCL2/CCR2 axis has proven difficult due to feedback mechanisms and the compensatory elevation of CCL2 104. In contrast, CCR2-targeted monocyte recruitment inhibition may prove more effective and durable than ligand-neutralizing strategies, but suppression will still be subject to that same limitation, among others 105. The CCL2/CCR2 axis is an attractive option to curb TAM recruitment. Further optimization will most likely be required to combine multiple strategies with better control of the size of the carriers, the targeting ligands, and the selectivity to the tissues.

Tumors that have high monocyte infiltration and rh chemokine-driven macrophages are the most appropriate target for recruitment-blocking strategies. The best part of these strategies is that they are pre-emptive and act before the accumulation of TAMs. These methods may not work as planned because of the redundancy of chemokines, recruitment of additional means, and an incomplete reduction of tissue-resident macrophages. Because of these factors, nanocarrier design would need to focus on tissue selectivity, sustained pathway stopping and coupling with immune checkpoint blockade or TAM reprogramming (or both), rather than a combined inhibition.

### 4.2 Depleting tumor-promoting TAMs

#### 4.2.1 Liposomal clodronate and bisphosphonate-containing nanocarriers as experimental TAM-depletion tools

This review considers bisphosphonates not as traditional anticancer drugs, but as macrophage-depleting agents or bone-affinity delivery vehicles used in some TAM-oriented nanomedicines. Bisphosphonates are commonly used in the clinical management of bone cancer associated with breast and prostate cancer with bone metastases 106. Some bisphosphonates are known to cause macrophage apoptosis and have been studied to target and reduce the population of tumor promoting macrophages 107.

Clodronate and zoledronic acid are the most studied bisphosphonates and anti-cancer drugs among them. Nevertheless, the use of unmodified bisphosphonates in the clinic as therapeutic agents is constrained by poor pharmacokinetics and a relatively high toxicity profile of renal function impairment, gastrointestinal distress, and osteonecrosis of the jaw 108. This risk-benefit ratio has prompted the design of bioinspired, nanocarrier-based bisphosphonate delivery systems aimed to improve macrophage targeting and bioavailability, while reducing toxicity.

Clodronate-loaded liposomes are the first example of macrophage depletion via the use of a bisphosphonate. Because of the liposomal particles’ macrophage-selective phagocytosis, the release of clodronate in their lysosomes can trigger apoptosis of the macrophages that degraded the liposomes. The effect of this method has inspired the use of combination therapies. Co-delivery of clodronate liposomes with doxorubicin liposomes was shown to reduce liposomal macrophage phagocytosis, enhance retention of doxorubicin, and improve its antitumor activity in a model of hepatocellular carcinoma 109. This approach demonstrates that the depletion of macrophages by bisphosphonates may also reduce the population of TAMs and improve the pharmacology and antitumor efficacy of the bisphosphonate-delivered anticancer drug.

To decrease the rapid systemic clearance and selective accumulation in the bone of bisphosphonates like zoledronic acid, formulations such as LipoZOL utilize liposomes, which help to mitigate rapid and extensive retention in the bone, allowing for more versatile applications of zoledronic acid beyond traditional bone-centric usages 110. Furthermore, the bone-targeting feature of zoledronic acid is exploited in the development of zoledronic acid surface-modified silica nanoparticles for the delivery of the chemotherapy agent doxorubicin, which is designed to provide a multifunctional therapy 111, 112. The presence of bisphosphonates in nanoparticles of this kind implies they could be used to deplete macrophages, and also function as dual-purpose carriers for the modulation of the tumor microenvironment.

However, several challenges are faced still regarding bisphosphonate-based TAM depletion. First, macrophage depletion by bisphosphonates is not exclusive to TAM and removes other macrophage populations as well 113. Second, bisphosphonates with a strong affinity for bone could be useful for targeting bone-metastasized tumors, but less for other cancers. Third, while reformulating bisphosphonates into nanocarriers improves delivery, it increases complexity and issues with translation beyond the lab. Overall, bisphosphonate-based nanomedicine is a promising approach to depleting macrophages, especially with tumors that contain bone-related pathology; however, improvements will be needed to provide a broader approach to utilize the system.

#### 4.2.2 Legumain-guided depletion of tumor-promoting TAMs

Legumain, an asparagine endopeptidase known to respond to hypoxia and stress, is an ideal candidate for selective tumor-associated macrophage (TAM)-based intervention. Legumain is found in tumor-associated macrophages, tumor-associated endothelial cells, and some tumor cells, but is mostly absent in normal tissues 114. Legumain’s favorable expression builds a strong case for use with trigger-responsive systems for drug delivery and tumor microenvironments for drug activation. The use of legumain for targeting is of interest for the biologically associated effects seen when legumain-positive TAMs are reduced. The mediators associated with legumain-positive TAMs and pro-tumor effects, such as TGF-β, TNF-α, MMP-9, and VEGF, have all been shown to contribute to angiogenesis, tumor growth, and metastasis. It is assumed that the reduction of TAMs corresponding to legumain would lead to a reduction of pro-tumor mediators, thus decreasing the effects of tumor growth and metastasis 115.

Based on this reasoning, active development of multiple legumain-responsive nanoplatforms aimed at selective activation and improved macrophage delivery-directed technologies was launched. One example could be the versatile system ATpep-NPs, where the author created the first multifunctional tool combining a phagocytosis-stimulating peptide and a legumain-cleavable substrate. Such systems enable conditional activation of the tool in legumain-overexpressing tumor tissues 116. Following the cleavage, the activated particles display an improved uptake and, via pathways related to Fc receptors, promote phagocytosis, which also facilitates TAM-oriented delivery and minimizes nonspecific interactions in the course of extended systemic circulation. In a similar manner, legumain cleavable peptide-drug conjugate nanoparticles PPP have been developed to enhance solubility of a drug, extend circulation, and achieve improved antitumor activity via tumor-selective activation 117. s-Tpep-NPs, a multifunctional system, utilizes legumain-responsive PEG shedding to transform a stealth nanoparticle system to an active form, enabling TAM targeting via phagocytic uptake mediated by Fc receptors 118.

The design of these systems and their physicochemical and biological properties affect their performance. Here, legumain not only works as a biomarker, but also as a tumor selective trigger for the activation of nanoparticles. Techniques such as cleavable linkers, depletion of PEG, and exposure of ligands, aim to reduce the uptake in circulation and increase activation in legumain abundant areas of the tumor. The effectiveness of these systems is determined by the cytotoxic payload and the design of the nanocarrier to the distribution of legumain, the macrophages, and the hypoxic regions of the tumor 119.

In general, legumain guided nanomedicine is a selective and ingenious method to target tumor-promoting TAMs. This isomer compared to non-selectively targeting macrophages, may result in better control of distribution and less influence on other phagocytic cell populations. Nonetheless, there are several challenges. Legumain is expressed in other TAMs, and may be different in other tumors and stages of the disease. Along with this, the therapeutic advantage of systems that are responsive to legumain is reliant on effective cleavage of the trigger, sufficient lag time in circulation before release, and consistency in performance of the formulation. Therefore, the key areas for improvement in the future will include the selectivity of the target, the sensitivity of the trigger, and easier manufacture *in vivo* 120.

#### 4.2.3 Selected macrophage-depleting strategies and their limitations

Along with bisphosphonate-based depletion and legumain-directed constructs, macrophage-depleting strategies have been investigated to further demonstrate the potential benefit of targeting tumor-supporting TAMs. Some of these strategies, unlike most of the trigger-responsive nanocarriers, focus on direct cytotoxicity against macrophages.

An example of one such strategy is trabectedin, which preferentially depletes monocytes and tumor-associated macrophages (TAMs) while having a relatively minor direct effect on some lymphocyte subsets, and therefore, has both a macrophage-depleting and anti-tumor action 121,122. In addition, a unique strategy involves the use of a bacterial system—attenuated Shigella flexneri—which is known to induce macrophage apoptosis 123. In a murine model of breast cancer, treatment with the attenuated Shigella flexneri resulted in a significant decrease in TAMs and complete tumor regression in the treated mice 124. These results show the significance of TAM depletion and how non-standard cytotoxic strategies can modify the tumor microenvironment.

On the other hand, and particularly when compared to more defined strategies of nanomedicine, these strategies do have serious limitations, such as relatively low target specificity, low biocompatibility, low control, and high standardization difficulties in a clinical context 125,126. Particularly, the use of bacterial vectors or broadly cytotoxic agents will have an effect on all macrophages, including those that reside outside of the tumor, and are unlikely to be incorporated into a targeted strategy of nanocarriers. Therefore, even though these strategies for TAM depletion are important for the development of the strategies’ early concepts, they will have much lower potential for the development and targeting of concepts compared to the other more flexible nanoplatform-based strategies.

### 4.3 Reprogramming TAM polarization and function

Macrophages are highly versatile. Within the tumor microenvironment, they can shift from an immune-supportive to a tumor-supportive role depending on the context. Accordingly, one important therapeutic strategy is not merely to deplete TAMs, but to reprogram their intrinsic phenotype and signaling state in a way that suppresses tumor progression and restores antitumor immunity. In this section, the emphasis is placed on nanomedicine strategies that primarily act by reshaping intrinsic TAM polarization, intracellular signaling, or microenvironment-responsive activation programs, rather than by directly restoring phagocytosis or serving mainly as delivery-enabling biomimetic platforms 127.

#### 4.3.1 Cytokine- and microenvironmental cue-mediated repolarization

One important route for TAM reprogramming is the modulation of cytokine-associated and microenvironmental cues that sustain the M2-like state. In tumors, macrophage polarization is strongly shaped by local mediators, including Th1- and Th2-related cytokines, redox imbalance, and nitric oxide signaling. Rather than simply inhibiting cytokine production, recent nanomedicine strategies have aimed to shift the local signaling context in favor of M1-like repolarization 128.

For example, HA-conjugated disulfide-linked polyethyleneimine-based nanoparticles (CPHT) have been reported to reduce the CD206/CD86 ratio and promote the upregulation of pro-inflammatory mediators such as TNF-α and iNOS, indicating a shift from M2-like to M1-like macrophage states. This effect is biologically relevant because increased nitric oxide production is associated with enhanced tumor-suppressive macrophage activity and reduced immunosuppression within the tumor microenvironment 129. Exogenous NO-delivery systems and S-nitrosothiol-modified dendritic mesoporous organosilica nanoparticles (DMON-SNO) utilize intracellular redox for controlled NO release. These systems increase CD80 and CD86 while decreasing CD206 and promote M1-like repolarization, by depleting glutathione and facilitating NO production 130.

The performance of these nanoplatforms depends on their physicochemical design. In CPHT, polymer assembly and ligand modification are used to improve delivery and modify macrophage-related biological responses. DMON-SNO incorporates a redox-responsive tetrasulfide framework that supports intracellular conversion of the NO donor. These examples highlight the advantage of assessing the design of the nanocarrier, as well as the bioactive molecule, to ensure effective TAM repolarization using the macrophage biology coupled with the tumor microenvironment 131.

Cytokine- and microenvironment cue-mediated TAM reprogramming is an innovative way to alter the tumor immune microenvironment without the need for macrophage elimination. However, this method is still limited by the inherent TAM heterogeneity, incomplete phenotype conversion, and the difficulty of achieving a balance between immune activation with an acceptable level of systemic inflammation.

#### 4.3.2 Modulation of NF-κB signaling in TAM reprogramming

NF-ĸB signaling plays a crucial role in macrophage activation and tumor-associated inflammation. It is challenging to categorize its role in TAM reprogramming as unambiguously pro-tumor or unambiguously anti-tumor as both the inhibition and activation of NF-ĸB pathways may change macrophage behavior in regard to the context in which they are triggered and the tumor environment 132.

An example of this paradigm was demonstrated in a study where NF-ĸB signaling was altered via miRNA modulation. Specifically, Wang *et al*. showed that the tumor progression of hepatocellular carcinoma was inhibited following the delivery of miR-99b to TAMs. In this study, the authors explained that miR-99b inhibited mTOR and ĸBRas2 and promoted differentiation of TAMs to the M1-like macrophage phenotype. In addition, TAMs that overexpressed miR-99b had improved phagocytosis and improved ability to present antigens. This study demonstrated that macrophage reprogramming is possible through the alteration of NF-ĸB signaling, and this paradigm is more advanced than simple modulation of cell surface markers 133.

In contrast, M1 repolarization is triggered by some nanomaterials through TLR/NF-ĸB pathway activation. An example of this is the use of graphene-based materials. The functionalized graphene oxide with PEG and PEI, when loaded with the CpG-Oligodeoxynucleotide, forms a complex that enhances pro-inflammatory immune activation and increases the immunostimulatory activity of CpG 134. In this case, TLR2 and TLR4 dependent NF-ĸB activation signaling induces M1 polarization and the production of pro-inflammatory factors 135. The response of these systems is influenced by the design and properties at the molecular and nanostructured level. Larger sheets of graphene oxide (750–1300 nm) were shown to promote M1 polarization more effectively than smaller sheets (50–300 nm) due to better interactions of larger sheets with TLR2 and TLR4 in the macrophage membranes, resulting in more effective NF-ĸB pathway signaling. This shows the effect of varying particle size on macrophage signaling and shows the necessity of understanding TAM biology when designing nanocarriers 136.

In general, when it comes to repolarizing TAMs, NF-ĸB modification shows a context-dependent approach. Its complexity limits its therapeutic applicability to repolar macrophages to more immune supportive functions. NF-ĸB and macrophage phenotype relationships are complex and not well understood and vary across different tumor types and their microenvironments. Excessive inflammation could cause damage to healthy tissue. Therefore, optimizing TACs in the future should consider the specificity of the signal, the degree of inflammation, and altering design of the TACs at the nanoscale, such as their size, surface features, and functionalized ligands.

#### 4.3.3 Mannose receptor-guided delivery for TAM repolarization

The mannose receptor (MR, also known as CD206) is widely recognized as a characteristic surface marker of M2-like macrophages and has therefore been extensively exploited for TAM-targeted delivery 137. In the context of nanomedicine, mannose-functionalized carriers provide a useful strategy for enriching the uptake of immunomodulatory cargos in tumor-promoting macrophages and thereby facilitating TAM repolarization 138.

A major advantage of MR-guided delivery is that it enables selective enrichment of therapeutic payloads in macrophage populations that are more closely associated with immunosuppression. Mannose-modified block copolymers have been shown to support targeted siRNA delivery and modulate macrophage-associated signaling pathways, including NF-κB-related immune activation, thereby promoting tumor cell apoptosis and immune remodeling 139. Likewise, comparative studies using hyaluronic acid-modified nanocapsules (HA NC) and hyaluronic acid/mannose dual-modified nanocapsules (HA-Man NC) demonstrated that incorporation of mannose significantly enhanced nanocapsule uptake by M2 macrophages and improved biodistribution in TAM-rich fibrosarcoma models 140. When HA-Man NC was further loaded with immunostimulatory cargos such as poly(I:C) and R848, robust antitumor activity and favorable safety profiles were observed in murine models of lung cancer and fibrosarcoma 141.

Guided by the mannose receptor, systems have been developed that not only deliver immune agonists but also augment the targeting of chemotherapeutics. For instance, doxorubicin-loaded mannose-conjugated bovine serum albumin nanoparticles (DOX@MAN-BSANP) demonstrated significantly greater endocytic uptake by M2 macrophages than by M1 macrophages, likely due to the mechanism of MR-mediated endocytosis. This preferential endocytic uptake indicates that, in certain contexts, drug carriers that are functionalized with mannose can be used to target both M2-like tumor-associated macrophages and tumor cells, thereby achieving dual macrophage modulation and endosomal delivery of cytotoxic drugs 142.

The fact that system performance depends on the selection of ligands and the design of the nanocarrier is also important. The density of mannose on the surface of a particle, the particle’s composition, and the presence of additional polymeric components (e.g., hyaluronic acid) can all affect macrophage recognition, cellular uptake, and tumor tissue distribution.

In summary, mannose receptor-mediated nanotechnology is an innovative and flexible tool for TAM-targeted delivery and TAM repolarization. Most importantly, MR/CD206 should be considered as a useful but not a completely TAM-specific marker, and its expression may not be the same in all tumors and/or macrophage states. Furthermore, significantly enhanced macrophage uptake is not likely to yield more prolonged and/or improved *in vivo* efficacy. Therefore, better addressing TAM heterogeneity and the macrophage functional plasticity in the tumor microenvironment will be important for future developments of targeted delivery systems.

#### 4.3.4 TLR agonist-based repolarization and immune activation

Recognized as important pattern recognition receptors in innate immunity, Toll-like receptors (TLRs) have various functions in macrophage activation and antitumor immune regulation. Concerning TAM-targeted nanomedicine, TLR4, TLR7/8 and TLR9 agonists are of great interest as they can drive the repolarization of pro-tumor, tumor-associated macrophages to a more pro-inflammatory state. They also promote innate-adaptive immune activation. Hence, TLR agonists are dual-purpose platforms that offer TAM reprogramming and immune enhancement 143.

A good example in this category is the R848-based nanoplatforms. Cyclodextrin nanoparticles that covalently incorporated the TLR7/8 agonist R848 (CDNP-R848) were developed by Rodell and colleagues. CDNP-R848 was seen to have an advantage of good interaction with phagocytic cells and also targeted M2-like TAMs in several tumor models. This system resulted in the remodeling of the TAM phenotype, the enhancement of anti-PD therapy, and the forming of tumor immune memory 144. In addition, the use of a nanoemulsion to deliver R848 was shown to maintain the agonist activity after freeze-drying and reconstitution, improve *in situ* immune activation and was even more effective when given together with tumor antigens. In murine tumor models, this method increased the recruitment of innate immune cells, promoted T-cell entry, modified the tumor-associated macrophages (TAMs) polarization state, and enhanced the activity of PD-1/PD-L1 blockade 145.

Nanovaccines have incorporated TLR agonists. For example, the multicomponent antitumor nanovaccine SVMAV used R848 in conjunction with a STAT3 inhibitor. This combination integrated innate immune stimulation with the inhibition of immunosuppressive signaling. After being administered, SVMAV preferentially targeted the lymph nodes, matured dendritic cells, enhanced antigen cross-presentation, and improved the cytotoxicity of CD8+ T cells against tumor cells. TLR agonist-based nanoplatforms will not only act on TAMs, but will likely also target numerous other immune compartments to modify the tumor microenvironment 146.

The design of the nanocarriers is critical to the functionality of the system. The composition of the carrier, the design of the cargo, and the distribution of the carrier all impact which myeloid population is activated by TLR. Furthermore, the stability of the formulation and the retention and release of the formulation also impact the level of systemic inflammation. Therefore, the design of the nanocarrier is important to meet the goal of immune activation and fragmentation along the desired cellular and anatomical locations 147.

Overall, TLR agonist-based nanomedicine is a novel approach to integrate TAM repolarization along with extensive antitumor immune activation. This approach does contain some limitations including: the potential for uncontrolled inflammation, the variability of immune responsiveness based on the type of tumor, and the challenge of balancing sufficient immune stimulation with systemic safety.

#### 4.3.5 PI3Kγ-, STAT-, and related pathways in TAM reprogramming

Beyond receptor-targeted and microenvironment-responsive strategies, intracellular signaling nodes provide another important route for TAM reprogramming. Among these, PI3Kγ and STAT3 are of particular interest because they function as central regulators of immunosuppressive myeloid programs and are closely linked to the maintenance of tumor-supportive macrophage states. Targeting these pathways may therefore shift TAMs toward a more inflammatory and immune-supportive phenotype while simultaneously alleviating broader myeloid-driven immune suppression 148.

PI3Kγ has been recognized as a key signaling node that promotes the suppressive phenotype of TAMs. Inhibition of this pathway can enhance inflammatory signaling and support M1-like repolarization. A representative nanomedicine example was reported by Li *et al*., who developed mannose-modified porous hollow iron oxide nanoparticles loaded with 3-methyladenine to improve TAM-directed delivery. In that system, carrier-mediated targeting together with PI3Kγ-related pathway inhibition activated NF-κB p65-associated inflammatory signaling in macrophages, promoted M2-to-M1 conversion, and inhibited tumor growth *in vivo* 149. The translational relevance of this axis is further supported by eganelisib (IPI-549), a first-in-class selective PI3Kγ inhibitor that demonstrated antitumor activity as monotherapy and in combination in the phase 1/1b MARIO-1 study 150.

STAT3 is an important intracellular regulator of tumor-associated macrophages. In tumor-associated macrophages (TAMs), STAT3 signaling is linked to immunosuppression, angiogenesis, and resistance to anti-tumor immunotherapies. Shobaki *et al*. used pH-sensitive CL4H6 lipid nanoparticles to deliver siRNA against STAT3 and HIF-1α, achieving selective uptake by TAMs, effective gene silencing, and suppression of pro-tumor programs linked to angiogenesis and immune suppression 151. In a more macrophage-selective design, CD163-targeted long-circulating liposomes carrying corosolic acid have been reported to preferentially inhibit phosphorylated STAT3 in CD163-positive monocytes/macrophages, illustrating how macrophage-enriched uptake can be combined with pathway-selective interference 15. Together, these studies indicate that STAT-directed nanomedicine can reshape macrophage function through more selective interference with immunosuppressive intracellular signaling.

Importantly, the performance of these systems depends not only on pathway selection but also on nanocarrier design. In this context, surface ligands such as mannose or CD163-targeting antibodies are used to enrich uptake in macrophage populations with tumor-supportive features, whereas pH-sensitive lipid systems can improve intracellular delivery and endosomal escape of nucleic acid cargo. PI3Kγ and STAT nanomedicine view intracellular signaling as an addition to selective carrier chemistries and TAM targeting within the tumor microenvironment. These methods are still limited by the pleiotropy of pathways, the type of tumor, and efficient reprogramming of macrophages without affecting the immune system.

PI3Kγ, STAT and other pathways are interesting method-based targets for reprogramming TAM because of their location on broad inflammatory, metabolic, and immunosuppressive pathways. Optimizing future efforts should prioritize the pathway in multifunctional systems and the selective delivery of these pathways and the targets TAM along with novel immunotherapeutic approaches.

#### 4.3.6 Emerging directions: efferocytosis-related macrophage regulation

Beyond receptor-targeted strategies and beyond microenvironment-responsive and intracellular signaling-based strategies, the regulation of efferocytosis-related macrophages is also emerging as a frontline strategy in the reprogramming of tumor-associated macrophages (TAMs). Efferocytosis, the phagocytic removal of apoptotic cells, is an essential process for the maintenance of tissue homeostasis and the resolution of inflammation. However, in cancer, this process can be used to support immune evasion and suppression 152. In tumor-associated macrophages, the frequent occurrence of efferocytosis is considered to be positively related to the signaling of M2 macrophages, anti-inflammation, and the inhibition of the cell-mediated immune response. For this reason, this process may represent a macrophage-related immune checkpoint in the tumor microenvironment 153.

In terms of mechanism, this process is most commonly described in the context of TAM receptors and the recognition of phosphatidylserine, with a strong focus on the receptor MerTK. MerTK is activated when ligands of GAS6 or protein S, which are involved in a bridge formation, allow phosphatidylserine of dying cells to be recognized by MerTK and subsequently signal macrophages to engulf apoptotic cells 154. Within the context of cancer, MerTK and efferocytosis are synonymous with immune evasion and the perpetuation of a macrophage tumor-supporting program. The experimental blockade of MerTK has been reported to diminish efferocytosis and promote antitumor immune response 155.

Recent studies further indicate that efferocytosis is not only a marker of macrophage function but also an active driver of tumor progression. In osteosarcoma, MerTK-mediated efferocytosis promoted immune tolerance and tumor progression by enhancing M2 polarization and PD-L1 expression 156. In bladder cancer, CD276-dependent efferocytosis by tumor-associated macrophages was shown to promote immune evasion 157. In pancreatic ductal adenocarcinoma liver metastasis, efferocytosis-mediated clearance of parenchymal dead cells reprogrammed macrophages toward an immunosuppressive state and facilitated metastatic outgrowth; pharmacologic or genetic interference with this pathway improved CD8⁺ T-cell function and reduced metastasis 158. Together, these findings support the idea that efferocytosis can directly reshape macrophage phenotype and the broader immune microenvironment.

Although still underrepresented in TAM-focused nanomedicine compared with pathways such as CSF-1R, TLRs, or PI3Kγ, efferocytosis-related targeting is beginning to enter nanoplatform design. A representative example was provided by Zhang *et al*., who developed tailored mesoporous silica nanoparticles to synchronize the release of doxorubicin and the efferocytosis inhibitor BMS777607, thereby coupling chemotherapy with blockade of MerTK-related apoptotic cell clearance 159. In this design, the nanocarrier was used not merely as a drug container, but as a means of coordinating cytotoxic tumor-cell killing with suppression of tumor-supportive efferocytosis, ultimately improving chemo-immunotherapeutic efficacy. This study is particularly important because it shows how pathway inhibition is not the only way to optimize efferocytosis-targeted tumor-associated macrophage (TAM) interventions using rational design of nanocarriers. Another example would be a system of glycopolymeric nanoparticles that preferentially deliver the MerTK inhibitor UNC2025 to tumor-associated macrophages in triple-negative breast cancer, which further illustrates the potential of merging both the blockade of efferocytosis and the design of macrophage-selective nanocarriers 160.

The biological and translational potential of this approach is the conceptual distinction from the simple design of macrophage depletion. Instead of the complete depletion of macrophages, the inhibition of tumor-supportive efferocytosis would prevent the clearance of apoptotic cells and the resultant immunosuppressive response, thereby changing macrophage behavior toward a less tolerogenic response 161. However, this also introduces new challenges. Since efferocytosis is a physiologically homeostatic process, excessive blockade may lead to the uncontrolled attainment of a state of tissue injury and inflammation due to an excessive buildup of necrotic cells. Additionally, many other receptors, in addition to MerTK, are involved in efferocytosis, including CD276 and various systems for the recognition of phosphatidylserine, thus providing multiple pathways and tumor-context dependence, which will most likely complicate the design of therapies 162.

Appraisal of macrophage regulation via efferocytosis is an original, valuable perspective for TAM reprogramming strategies. While the previous sections concentrated on disruption through receptor blockade, inflammatory pathway agonism, or blockade of intracellular pathways, this perspective demonstrates how clearance of dead cells shapes macrophage phenotype and mediates immune evasion. For this perspective to be useful, targeting tumor-associated macrophages should be more selective. The distinction between blockade: helpful versus harmful, as well as the integration of efferocytosis within the framework of nanocarriers, should be addressed. This includes the design of the size, surface chemistry, trigger and responsiveness as well as chemistry of the nanoparticle and selective uptake by macrophages 163.

Efferocytosis-based TAM reprogramming strategies are appealing because they maintain the abundance of macrophages and redirect their function to supportive, immune-promoting states. However, this often results in a more pronounced phenotype that is less stable and more susceptible to the dominant tumor-derived, suppressive signals. This demands more precise control over the design of nanocarriers to achieve the active and directed delivery of inflammatory signals and greater alignment with the degree of plasticity dominantly expressed by macrophages in each tumor context.

### 4.4 Restoring macrophage phagocytic and immune-activating functions

The strategies covered in this section differ from the reprogramming strategies discussed in Section 4.3. The strategies in Section 4.3 focus on modifying the internal state of the TAMs as opposed to the strategies described in this section. These strategies focus on restoring output, in particular enhancing the tumor cell uptake, antigen recognition, and crosstalk of adaptive and innate immunity. Therefore, the core logic of this section is not broad phenotypic repolarization, but functional reactivation of macrophage-mediated antitumor immunity through phagocytic checkpoint blockade, immune co-stimulation, or cell engineering 164.

#### 4.4.1 Blocking the CD47/SIRPα axis

The CD47/SIRPα axis is one of the best-characterized antiphagocytic checkpoints in cancer. Signal regulatory protein α (SIRPα), expressed on macrophages and other phagocytic cells, binds to CD47 on tumor cells and transmits a “don’t eat me” signal that suppresses phagocytosis. In many preclinical tumor models, blockade of this pathway restores macrophage-mediated engulfment of tumor cells and enhances antitumor immunity. Accordingly, disruption of CD47/SIRPα signaling has emerged as a central strategy for reactivating macrophage effector function in the tumor microenvironment 165.

Although anti-CD47 antibodies remain the most common approach to interrupt this pathway, recent studies indicate that CD47/SIRPα blockade is often more effective when integrated into multifunctional or combination-based therapeutic designs rather than used alone 166. For example, the CD47 blocker SIRPα-Fc has been reported to enhance the antitumor efficacy of CAR-T cells, suggesting that interruption of the “don’t eat me” signal may cooperate with T-cell-directed strategies to strengthen overall immune attack against tumors 167. This observation is consistent with the broader concept that restoration of macrophage phagocytosis can complement adaptive immune activation.

Nanomedicine-based formulations have been developed to improve the spatiotemporal control of CD47/SIRPα blockade. One representative example is the use of M1 macrophage-derived exosomes conjugated with CD47 and SIRPα antibodies. In this design, the mildly acidic tumor microenvironment triggers antibody release, thereby blocking CD47/SIRPα signaling while the M1-derived exosomal carrier simultaneously promotes macrophage repolarization toward a more pro-inflammatory phenotype. As a result, this platform combines checkpoint disruption with macrophage reprogramming and enhanced tumor cell clearance 168. Similarly, ROS-responsive albumin nanoparticles co-loaded with PD-1 and CD47 antibodies have been used to couple T-cell activation with restoration of macrophage phagocytosis. Upon exposure to intratumoral ROS, these nanoparticles release anti-PD-1 to activate T cells and anti-CD47 to enhance macrophage-mediated tumor cell engulfment and antigen presentation, thereby amplifying antitumor immune responses *in vivo* 169.

A related but conceptually distinct strategy is to combine blockade of “don’t eat me” signaling with reinforcement of “eat me” signals. An example of a dual-signal strategy was reported by Luo *et al*. They created a nanoparticle system to block CD47-SIRPα signaling and increase calreticulin. This system showed greater efficacy in macrophage-mediated tumor-cell clearance and enhanced the efficacy of chemo-immunotherapy by using a combination of signal suppression of the antiphagocytic signaling pathway and enhancement of the pro-phagocytic signal. This study is a good example of the application of a design of a nanocarrier to integrate both the inhibitory and the stimulatory signals for the phagocytic process, rather than modify one signal at a time 170.

The design of the nanocarrier is crucial for the functioning of the systems in addition to the choice of antibodies. For example, the use of pH-sensitive systems can confine the release to the acidic regions of the tumor, while ROS-responsive carriers can couple the activation to oxidative stress. Furthermore, exosome- or albumin-based systems may improve circulation, biocompatibility, and immune system modulation. For successful CD47/SIRPα-directed systems, the design of the nanocarriers must integrate the aforementioned concepts 171.

In general, the current design of systems to block the CD47/SIRPα pathway may improve the ability of macrophages to perform phagocytosis and enhance the antitumor immune response. This system, however, faces a number of problems that must be taken into consideration. These include balancing immune safety and the CD47 expression system on other normal cells, which limits the control of immune responses. Optimizing design for systems in the tumors Defensive Design systems and Rational Design systems may help address these problems 172.

#### 4.4.2 CD40-mediated enhancement of antigen presentation and innate–adaptive immune crosstalk

CD40 is a member of the TNF receptor superfamily and is broadly expressed on antigen-presenting cells, including dendritic cells, macrophages, and B cells. Engagement of CD40 by CD40L promotes antigen presentation, upregulation of MHC molecules, and secretion of pro-inflammatory mediators, thereby strengthening T-cell priming and adaptive immune activation. In the context of tumor immunotherapy, CD40 signaling is particularly important because it links myeloid-cell activation with the generation of effective antitumor T-cell responses 173. Accordingly, CD40-oriented systems are discussed here not as general TAM-reprogramming approaches, but as strategies that enhance the immune-output functions of macrophages and related antigen-presenting cells.

In this context, CD40-directed nanomedicine should be understood not simply as a macrophage-targeting strategy, but as an approach that amplifies innate–adaptive immune crosstalk. A representative example was reported by Yan *et al*., who developed R848-derived amino lipids and incorporated them into lipid nanoparticles for delivery of CD40 mRNA to dendritic cells. In that system, the lipid nanoparticle formulation readily transfected CD40 mRNA. In combination with agonistic anti-CD40 antibodies, this formulation produced impressive antitumor effects in a murine melanoma system, including inhibition of tumor growth, enhancement of survival, and generation of immune memory. This example is noteworthy because it demonstrates that the integration of innate immune agonism with the use of nanocarriers for the delivery of nucleic acids, and the enhancement of antigen presentation, also has the potential to increase T-cell responses mediated by CD40 174.

From a mechanistic point of view, this example also complements the previous subsection focused on CD47/SIRPα blockade. Here, CD47/SIRPα inhibition would allow for the engulfment of tumor cells by macrophages. Conversely, CD40 would provide a more potent effect at the level of antigen presentation and would serve to amplify the immune response. For this reason, the CD40-targeted approaches would provide an immune-extensive effect rather than a phagocytosis-restorative effect.

In this regard as well, there is evidence outside the realm of nanomedicine that supports the significance of CD40 in biology. For example, in certain models of hepatocellular carcinoma that are associated with non-alcoholic fatty liver disease, it has been shown that CD40L derived from platelets contributes to the activation of CD8+ T-cells and suggests that the CD40 pathway of immune system activation may play a role in anti-tumor immunity within other inflammatory settings as well 175.

The functioning and effectiveness of CD40-targeted systems also relies on the design of the carriers and not just the agonist. As such factors as the lipid formulation in the carrier, the efficiency with which mRNA is encapsulated, how it is delivered, and the tissue localization, all influence whether CD40 is activated in dendritic cells, macrophages or other antigen presenting cells in the body 176. CD40-oriented nanomedicine requires wise and meticulous design to achieve a balance that maximizes the effect of immune activation while minimizing harmful and systemic inflammatory effects 177.

CD40 is a potentially powerful tool for enhancing antigen exposure and bolstering anti-tumor T-cell activity. Its broad distribution within the immune system can lead to increased inflammation and unintended immune responses. Future refinements of this approach are likely best directed at increasing selective delivery. Changes that combine CD40 targeting with T-cell or macrophage focused approaches are also likely to enhance the balance of immune response with the systemic tolerability of the effect 178.

#### 4.4.3 Chimeric antigen receptor macrophages (CAR-M)

Chimeric antigen receptor macrophages (CAR-M) are an innovative cell engineering method formed to bolster macrophage antigen-specific phagocytosis and instigation of tumor-directed immune response. Unlike the previously discussed nanocarrier-based delivery systems, CAR-M therapy focuses on the direct modification of macrophages, whether it be *ex vivo* or *in situ*, to help recognize tumor-associated antigens and enable proficient tumor cell clearance. For these reasons, CAR-M can be deemed a complementary method of TAM-targeted therapy, integrating macrophage-centric phagocytosis and immune restructuring 179.

The first adaptation of CAR-M was established by Klichinsky *et al*., who created HER2-targeting CAR macrophages, which demonstrated a powerful antitumor effect both in vitro and *in vivo*. CAR-M not only increased tumor cell phagocytosis, but also enhanced the tumor microenvironment’s immunosuppressive signaling resistance, along with the production of pro-inflammatory cytokines and antigen presentation. These findings indicate that CAR-M have the potential to operate not only as tumor-clearing phagocytes, but as modulators of the immune microenvironment as well 180.

Later studies built on this idea. Chen *et al*. showed CAR macrophages could target HER2 and CD47 and demonstrated specific antigen phagocytosis of ovarian cancer cells that also activated CD8⁺ T-cells 181. This design is important conceptually because it integrates specific antigen recognition and removal of macrophage anti-phagocytosis blockade, which reinforces both the engulfing of the tumor cell and the activation of the adaptive immune system. In this regard, CAR-M therapy is related to what was previously described concerning the blockade of the CD47/SIRPα, but is more sophisticated and cell-centered concerning the macrophage-mediated tumor eradication approach. In the case of postoperative glioblastoma, an important advance to the CAR-M concept was the intracavitary production of glioma stem cell-targeting CAR macrophages to address residual tumor and stimulate local immunity. In this regard, this study is especially interesting because it shows that CAR-M can be applied to anatomically confined and clinically difficult approaches, expanding the translational possibilities of macrophage engineering beyond typical systemic administration 182. This study also suggests that future designs of CAR-M may especially benefit from implementation in an anatomically and clinically reasonable context and not just general systemic administration.

Kang *et al*. formulated MPEI/pCAR-IFN-γ nanocomplexes using mannose-conjugated polyethyleneimine for the delivery of plasmid DNA for ALK-specific CAR and IFN-γ. The nanocomplexes transfected tumor microenvironment-associated macrophages and assisted the development of CAR-M1 cells that possess tumor antigen-specific phagocytosis. Furthermore, the system promoted the upregulation of M1 markers and downregulation of M2 markers, suggesting the combination of CAR phagocytosis and macrophage repolarization can occur within the same therapeutic framework. This example is particularly applicable to the current review, as it intersects cell engineering and nanomedicine, and demonstrates that macrophage programming can be achieved using a carrier design approach rather than relying solely on adoptive transfer 183.

Even with the benefits that CAR-M provides, it is still a labor-intensive approach. Manufacturing of CAR-M is much more complicated compared with the modulation of macrophages using antibodies or nanoparticles. In addition, the modulation of CAR-M is much more controlling, and a greater emphasis must be placed on its persistence, biodistribution, and safety. Furthermore, the variability of macrophrophage populations, as well as the antigen expression which is dependent on the tumor type, and the microenvironment of the tumor, which is immunosuppressive, may impact the efficacy of CAR-M *in vivo* 184. In total, TAM-targeted therapies greatly benefit from CAR-M, but the scalability of CAR-M will also rely on various controllable integrations with macrophage-focused strategies and checkpoint blockades 185.

Phagocytosis-restoring and immune-output-enhancing strategies work by directly restoring macrophage epithelial functions and effector functions rather than simply changing the polarization markers. They help integrate the processes of tumor cell engulfment, antigen presentation, and the activation of the adaptive immune system. Nevertheless, safety issues associated with systemic immune activation, widely heterogeneous antigens, and off-tumor immune responses arise from the previously mentioned activities. Future designs of these strategies should focus on activating the immune system exclusively in the tumor, the strategic combination of these approaches with checkpoint blockade, and the careful assessment of macrophage-mediated antigen presentation in the immune system, as opposed to the phagocytosis.

### 4.5 Biomimetic and macrophage-derived delivery platforms

In contrast to the mechanistically defined intervention classes discussed above, this section focuses on macrophage-derived or macrophage-mimetic systems as delivery-enabling platforms. These platforms should not be regarded as a separate macrophage-targeting mechanism equivalent to recruitment blockade, depletion, or reprogramming. Instead, their value lies in improving circulation behavior, inflammatory homing, payload protection, spatial precision, and microenvironment-responsive delivery 186. Accordingly, biomimetic platforms can be combined with multiple TAM-oriented mechanisms, including macrophage depletion, repolarization, phagocytosis restoration, and immune co-stimulation. Recent studies further suggest that biomimetic design is evolving from passive membrane camouflage toward actively engineered macrophage-directed immune modulation 187,188.

#### 4.5.1 Macrophage membrane-coated nanoparticles

Macrophage membrane-coated nanoparticles represent a biomimetic delivery strategy that takes advantage of the inflammatory tropism and immune-interface properties of macrophage membranes. Rather than constituting a separate mechanistic class of TAM intervention, these systems are better understood as engineering platforms that can improve circulation behavior, reduce premature clearance, and enhance delivery precision in tumor or inflammation-associated sites through membrane-associated surface proteins 189. In this sense, macrophage membrane coating should be viewed primarily as a delivery-enabling strategy that can be integrated with multiple therapeutic payloads and TAM-modulating mechanisms.

A representative example was reported by Huang *et al*., who developed cRGD-modified macrophage membrane-coated nanovesicles for co-delivery of METTL14 and a TLR4 agonist. In that study, the macrophage membrane supported dual targeting toward tumors and macrophages, while cRGD further enhanced tumor-associated targeting. The formulation was designed not only to inhibit tumor growth but also to promote macrophage polarization toward an M1-like phenotype, illustrating how macrophage membrane coating can be combined with immunomodulatory cargo design rather than serving as passive camouflage alone 190.

Macrophage membrane camouflage has also been used to improve recruitment to inflammatory tumor sites and to support combination therapy. Zhao *et al*. reported quercetin-loaded hollow bismuth selenide nanoparticles camouflaged with macrophage membranes, in which CCL2-associated recruitment and hyperthermia-triggered drug release were integrated to enhance photothermal sensitivity and antitumor efficacy. This example shows that rather than simply prolonging circulation, macrophage membrane coating may interact with chemokine-responsive tumor biology 191.

Moreover, Hybrid membrane systems are an extension of this idea. For glioma therapy, Yin *et al*. innovated a dual membrane coating of macrophages and neutrophils around rapamycin PLGA (poly(lactic-co-glycolic acid)) based nanoparticles (NMm-PLGA/RAPA). The active hybrid membrane of this system provided the carrier with both macrophage-based homing and neutrophil-based inflammatory chemotaxis, thus enabling barrier crossing, tumor targeting, and sustained glioma regression *in vivo*. This work supports the idea in this section that macrophage membrane systems may be functionally combined with other membrane systems to address different pathological challenges of trafficking and barrier crossing 192.

Finally, even the therapeutic benefits of macrophage membrane-coated nanoparticles are determined by the drug and design elements such as the integrity of the membrane, size, membrane-core, and possible additional ligands and membrane proteins. These design features will affect the tumor-site localization, the uptake by cells, and the biodistribution. Thus, using a macrophage membrane coating is not a simple surface adornment of a polymer carrier, but a sophisticated and biologically relevant engineering design approach 193.

Macrophage membrane-coated nanoparticles offer an innovative approach to drug delivery systems in oncology. to date, the delivery system faces membranes-related standardization, production scalability and control, safety and efficacy aspects, and high variability in the delivery system. Optimizing the technology needs to consider the design of the membranes, controlled delivery, and design of the surface of the nanoparticles for a specific therapeutic payload to treat specific tumors 194.

#### 4.5.2 Macrophage-derived and engineered exosome platforms

Exosomes are small extracellular vesicles that can carry a variety of biological molecules. They are found in most biological fluids and are gaining attention as novel drug delivery vehicles for cancer therapy 195. With regard to macrophage-based systems, it is imperative to differentiate between naturally immunosuppressive TAM/M2-derived exosomes that promote tumor progression and drug resistance, and exosomes that are therapeutically helpful and may be derived from engineered macrophages. Current therapeutic approaches have focused on exosomes derived from M1-polarized macrophages, or on engineered systems of macrophage-derived exosomes that allow for precision in modulation of exosomal cargo and targeting 196.

An example of this is the system of M1 macrophage-derived exosomes that are loaded with docetaxel, as reported by Zhao *et al*. In this study, docetaxel was loaded to produce DTX-M1-Exo, that not only delivered docetaxel to the tumors, but also modulated and sustained an M1-like inflammatory response in an otherwise immunosuppressive tumor microenvironment. The system evidenced a higher potency in the inhibition of tumor growth, as compared to the free drug or non-exosomal systems in breast cancer, demonstrating that exosomes can act as drug carriers and immune-response modulators 197.

Gene and combination delivery systems have also been developed using exosomes derived from macrophages. Miao *et al*. used M1 macrophage-derived extracellular vesicles carrying siCX3CR1 for treatment of pancreatic cancer. This method, utilizing M1 EVs for delivery of nucleic acids, was based on targeting the CX3CL1–CX3CR1 axis and was able to inhibit the proliferation and migration of AsPC-1 cells and reduce the growth of the tumors *in vivo* 198. In a more recent example, Xu *et al*. mixed cationic liposomes and macrophage-derived exosomes and proposed a system to co-deliver docetaxel and Bcl-2 siRNA that was made to respond to the presence of reactive oxygen species (ROS). The design was evaluated for its targeting capabilities and its safety profile in a breast cancer system, and it was considered to be both efficacious and enhanced for the controlled delivery of the siRNA and the chemotherapeutic 199. The work of these groups demonstrates that the systems derived from macrophage exosomes can be engineered to improve the control of how the system releases its contents and also to improve the versatility of the system’s contents.

Therapeutic extensions of macrophage-derived exosomes also focus on engineering modifications. Jiang *et al*. created a CAR-M-derived exosome-drug conjugate (CAR-EDC) using a pH-sensitive linker to attach the drug SN-38 to exosomes derived from CAR macrophages that had been engineered to express the CAR specific for the CD19 antigen. In this study, the CAR modular component was responsible for the selective uptake of the conjugate by CD19+ tumor cells. In addition, the exosome component enhanced the immune response due to the enrichment of CXCL10. This example is pivotal for this particular area as it integrates exosome engineering, tumor targeting, and the design of a system to combine chemotherapy with immunotherapy, all within a single biomimetic approach 200.

Therapeutic performance of macrophage-derived exosome systems will depend on other elements apart from carrier selection, including design parameters like choice of vesicle source, polarization of the donor macrophages, membrane composition, loading method, hybridization method, and the choice of a trigger for release control. Macrophage-derived exosomes should not be considered as naturally targeting vesicles, but as biologically guided delivery systems whose function depends on the extent to which exosome engineering is aligned to macrophage biology and the tumor context 201. However, significant challenges must be addressed, including the heterogeneity of exosome content, difficulties in large scale isolation and quality control of exosomes, and the possible transmission of exosome-mediated protumor signals due to poorly characterized macrophage-derived vesicles 202.

Macrophage-derived and engineered exosome platforms are a promising extension of biomimetic nanomedicine for oncology. Exosomes, as compared to macrophage membrane-coated nanoparticles, have flexible engineering and intrinsic cargo capacity. However, Exosomes pose even larger challenges in standardization and in determining mechanisms of action 203. Future optimizations should focus on high design control, defined engineering with functional differentiation of native tumor-associated macrophage exosomes and of designer macrophage-derived exosomes.

The biomimetic systems in this section should be thought of more as modular delivery systems and less as additional mechanism-specific therapies. These systems can improve the spatial precision and translational versatility of a variety of tumor-associated macrophage-targeted therapies. In Table 3, we capture an overview of TAM-targeted approaches and compare carrier systems using a variety of design and engineering parameters, TAM biology rationale, therapeutic effects, translational gaps, and current stage of development.

### 4.6 Clinical translation and clinical trials

While the earlier sections describe many therapeutic strategies focused on tumor-associated macrophages (TAM), their translational maturity is very disparate. Currently, the most advanced clinical data are associated with antibody, small molecule, and cell-based immunotherapy designed around specific pathways, while the majority of multifunctional, TAM-targeting, nanoplatforms and biomimetic delivery systems are still in the preclinical stage. This disparity represents a larger problem for the field of nanomedicine, where a strong biological rationale has to be complemented through robust characterization, manufacturability, and safety, as well as clinical benchmarking and biodistribution 204.

#### 4.6.1 Current clinical candidates and trial progress

The current clinical landscape shows a clear maturity gradient. Pathway-directed small molecules and antibodies, such as CSF1R, CCR2, CD47/SIRPα, and PI3Kγ inhibitors, have advanced further because their pharmacology, manufacturing, dosing, and safety assessment are relatively more standardized. By contrast, multifunctional TAM-targeted nanoplatforms face additional translational barriers, including complex composition, batch-to-batch variability, unclear biodistribution, and limited clinical benchmarking. Therefore, current clinical candidates should be interpreted not only as therapeutic examples, but also as benchmarks for evaluating the translational readiness of emerging TAM-oriented nanomedicines. Among currently available TAM-directed approaches, the most clinically mature candidates are still concentrated in pathway-targeting antibodies, small molecules, and cell-based immunotherapies rather than in complex nanoplatforms. The representative clinical directions discussed below illustrate both the therapeutic promise and the current limitations of macrophage-oriented intervention in cancer 205.

Among recruitment- and macrophage-depletion-oriented strategies, the CSF1/CSF1R axis currently has the strongest clinical precedent. Pexidartinib, a CSF1R inhibitor, is FDA-approved for symptomatic tenosynovial giant cell tumor (TGCT), providing proof that macrophage-directed CSF1R inhibition can be clinically actionable 206. In advanced solid tumors, emactuzumab showed pharmacodynamic depletion of immunosuppressive M2-like macrophages in a phase I study 207, and LY3022855 was evaluated in combination with durvalumab or tremelimumab in a phase 1a/1b trial, although clinical activity in solid tumors remained limited 208. Together, these studies suggest that CSF1R-directed therapy is clinically tractable, but that success in macrophage-rich solid tumors may require more effective patient selection or rational combinations.

The CCL2/CCR2 axis has also advanced into clinical testing, particularly in pancreatic cancer, where inflammatory monocyte recruitment is a prominent component of the tumor microenvironment. PF-04136309 reached phase Ib evaluation with FOLFIRINOX and with gemcitabine/nab-paclitaxel, showing a significant clinical proof-of-concept for blockade of CCR2-mediated macrophage recruitment 209,210. However, these studies also show a larger issue for the class, which is that while these provide some evidence of activity, the benefits are not durable and will not likely become an accepted part of a clinical regimen.

Of the phagocytosis-restoring strategies, blockade of the CD47/SIRPα pathway is currently the most clinically advanced. In solid tumors, magrolimab-containing combinations are progressing to phase 1b/2, with example of magrolimab plus cetuximab showing tolerability and evidence of antitumor activity in heavily treated colorectal cancer and other solid tumors 211. These results support the translational relevance of macrophage checkpoint blockade, but they also indicate that CD47-directed therapy is more likely to succeed in combination settings than as a stand-alone intervention.

For macrophage reprogramming beyond surface checkpoint blockade, PI3Kγ inhibition has also entered the clinic. Eganelisib (IPI-549), a first-in-class selective PI3Kγ inhibitor, demonstrated manageable safety and antitumor activity as monotherapy and in combination with nivolumab in the phase 1/1b MARIO-1 study. This is notable because it clinically validates the concept that reprogramming suppressive myeloid states, rather than only depleting macrophages, may be therapeutically meaningful in solid tumors 212.

Finally, the cell-engineering branch of TAM-targeted therapy has begun to reach first-in-human testing. CT-0508, an anti-HER2 CAR-macrophage product, entered phase I evaluation in HER2-overexpressing solid tumors 213, and interim clinical results demonstrated feasibility, tolerability, tumor trafficking, and evidence of tumor microenvironment remodeling 214.

Taken together, current clinical candidates indicate that TAM-directed therapy has moved beyond conceptual promise, but remains at an early and uneven translational stage. The most clinically mature approaches still target macrophage recruitment, survival, phagocytic checkpoints, or intracellular signaling through antibodies, small molecules, or cell therapy, whereas the more sophisticated nanoplatforms discussed in earlier sections have not yet generated comparable clinical datasets. This contrast directly raises the question of why biologically compelling TAM-oriented nanomedicines have translated more slowly, which is addressed in the following section.

#### 4.6.2 Key translational barriers: specificity, penetration, safety, and manufacturability

Despite the compelling biological rationale and encouraging preclinical activity summarized above, the clinical translation of TAM-oriented nanomedicine remains constrained by several recurring barriers. Across the broader nanomedicine field, successful translation depends not only on proof-of-concept efficacy but also on whether a platform can achieve reproducible targeting, adequate tumor delivery, acceptable safety, and scalable manufacturing under clinically relevant conditions 215. These constraints are especially important for macrophage-targeted systems because they must operate within a heterogeneous immune microenvironment while also avoiding premature clearance and unintended immune perturbation 216. Recent work has further suggested that improving translation may require not only better systemic nanocarriers but also more anatomically and clinically tailored delivery formats. For example, Kaiser *et al*. described a biodegradable implant capable of sustained release of immune-modulatory small molecules to reprogram immunosuppressive myeloid cells in the surgical cavity after glioblastoma resection. Preclinically, this method increased interleukin-12 expression in myeloid cells with no systemic cytokine increase and improved T-cell infiltration. This shows that myeloid-centric strategies can be combined with local postoperative therapies to design more clinically applicable treatment strategies 217.

The first major hurdle is specificity. Although many TAM-targeted strategies utilize CD206 or CD163, those markers correspond to subsets of macrophages that are not tumor-associated and are rather immunosuppressive. Their expression may change a lot from tumor to tumor and even from stage to stage and context to context in a given disease. It is further noted that TAM subsets that express CD206, CD163, MARCO, and TREM2 have varied functions and prognostic roles, demonstrating that “TAM targeting” is quite rarely a single-population issue 218. Consequently, ligand-mediated delivery approaches often achieve preferential uptake, rather than absolute target selectivity, which may explain why promising preclinical targeting efforts often yield disappointing results when applied *in vivo* 219.

The second major hurdle is tumor penetration and the distribution of agents within the tumor. Even when delivery is accomplished systemically, a stiff and dense ECM combined with the highly compressed tumor stroma, aberrant angiogenesis, and high interstitial fluid pressure can limit the extravasation, diffusion, and impenetrable tissue to nanoparticles. This is especially evident with stromal-rich tumors, but applies similarly to most solid tumors where macrophages are not evenly dispersed but are concentrated in perivascular, hypoxic, or fibrotic niches 220. Because of this, a carrier that is taken up by peripheral myeloid cells or that is delivered to the tumor margin may still be unable to access the tumor regions where the bulk of macrophs reside and where the most impenetrable macrophs are likely located.

The third barrier is safety and biological unpredictability. After nanoparticles are introduced into body fluids, they quickly develop a protein corona, and their biological identity may be altered. This may impair their target recognition, significantly change their biodistribution, and consequently influence cellular uptake and drug release. Additionally, the rapid capture of nanoparticles by the mononuclear phagocyte system (MPS) will greatly diminish the number of nanoparticles available to be delivered to the tumor 221. This is very problematic for TAM-oriented systems because macrophages are both the target of the therapy and a major sink for the off-target uptake. Therefore, approaches to enhance the interaction of macrophages at the tumor site may lead to increased clearance in the liver, spleen, and other tissues rich in macrophages, which may limit the therapeutic margin and further worsen the safety evaluation of the system 222.

The fourth barrier, manufacturability and quality control. Problems in this area start to become evident, especially for biomimetic systems such as membrane coated nanoparticles, macrophage-based carriers, and platforms based on extracellular vesicles or exosomes. Recent translational reviews indicate that large-scale production, traceability, product homogeneity and purity, stability, and GMP compliance are still unresolved for many advanced formulations 223. In systems based on macrophages and membrane systems, even more challenges result from variability of donor-cells, reproducibility of membrane isolation and assembly, immune compatibility, and control of drug loading and release. These problems are not secondary engineering details, but they are the main factors that will determine whether a system will move from preclinical work to clinical testing 224.

Combined, these barriers illustrate why the most advanced TAM-targeting methods in the clinic are limited to antibodies, small molecules, and cell therapies, while many advanced nanoplatforms reside in the preclinical space. Progressing the field may demand a more robust translational framework that accounts target biology, carrier design, and the concepts of pharmacology, safety, and manufacture from the beginning, not as an afterthought or in a sequential manner. In regard to TAM-targeted nanomedicine, the advancement of the field will rely on the ability to more precisely target subsets, develop novel strategies to access the core of tumors, control off-target macrophage uptake, and incorporate manufacturability and quality control in an earlier design of the platforms. Several clinical candidates, barriers, and future opportunities are summarized comparatively in Table 4 and are discussed in more detail in the following section.

## 5. Discussion and Perspectives

**Figure 4:**
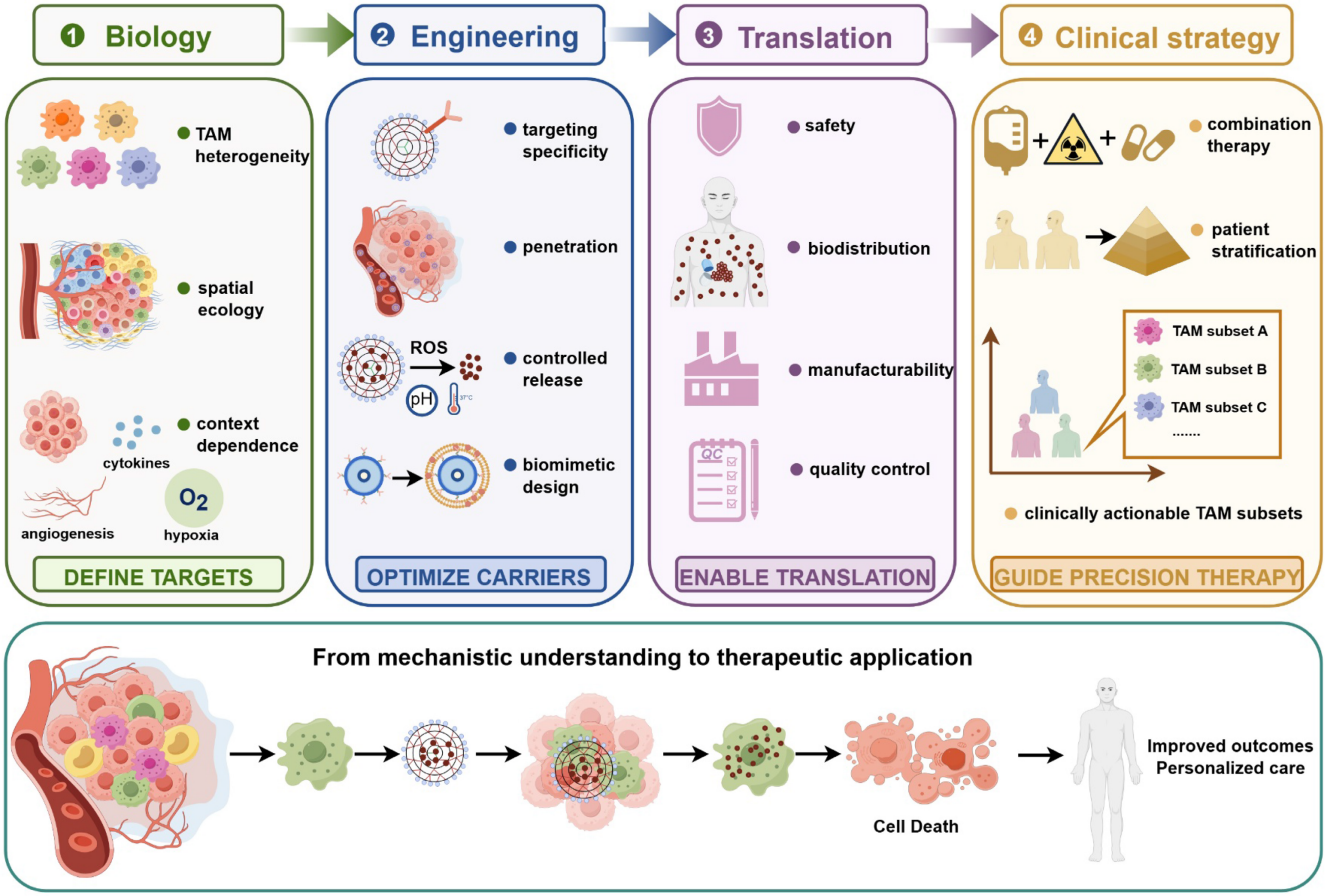
** Translational roadmap for TAM-targeted nanomedicine.** Stepwise integration of macrophage biology, nanocarrier design, translational research, and clinical planning will be needed for the clinical progression of tumor-associated macrophage (TAM)-targeted nanomedicine. In the first step, TAM heterogeneity and spatial ecology, as well as context-driven cytokine signaling, angiogenesis, and hypoxia, help determine relevant therapeutic targets for TAMs and functional subsets. In the second step, the design of nanocarriers focuses on the delivery efficiency of TAM-targeted nanomedicine, incorporating innovative features such as design of the carriers using stimuli-responsive strategies, enhanced penetration across tumors, and greater specificity for tumor-associated TAMs. Third, translational evaluation must address major barriers, including safety, biodistribution, manufacturability, and quality control, which determine whether preclinical nanoplatforms can be advanced toward clinical testing. Finally, clinical strategy should integrate rational combination therapy, patient stratification, and identification of clinically actionable TAM subsets to guide precision therapy. The lower panel illustrates the conceptual progression from mechanistic understanding of the TAM-rich tumor microenvironment to carrier optimization, tumor delivery, macrophage modulation, tumor cell killing, and ultimately improved outcomes and personalized care.

Recently, tumor-associated macrophages (TAMs) have been recognized as crucial factors driving tumor progression, remodeling immunity, and creating resistance to therapy. As such, they present both alluring and intricate targets for nanomedicine interventions. As we have articulated, the prevailing question is no longer ‘can we target TAMs?’. Rather, we must understand the precise and biologically relevant therapeutic outcomes necessary for clinical applicability. This will demand a thorough understanding of the TAMs’ heterogeneity, the tumor microenvironment, and how we design the drug delivery systems themselves. Thus, in a broad sense, TAM nano-interventions are an assemblage of targeting frameworks and nanomedicine constructs. More specifically, and in a concerted manner, they are an integration of the biology of macrophages, advanced engineered nanocarriers, and translational oncological research 225. To further illustrate these frameworks, Figure 4 represents a translational pathway outlining potential TAMs interventions from nanotechnology and engineering design to clinical research and practice.

A major advancement in the DM field is the acknowledgment of the heterogeneity of tumor-associated macrophages (TAMs). There is growing evidence from single-cell RNA sequencing, spatial transcriptomics, and advanced profiling that TAMs show significant heterogeneity across different tumor types, stages of disease, TAMs location in the body, and the context of treatment 226. This heterogeneous nature would indicate that TAM-targeted nanomedicines would be better served targeting specific macrophage subsets and not generally targeting ‘macrophages’ 227. Therefore, addressing these issues would likely result in the better integration of the macrophage, spatial tumor microenvironment, and design of delivery systems.

A second central problem is that many of the strategies currently available are still inadequate when used alone. Recruitment blockade, direct depletion, and intracellular reshaping, among others, target only certain aspects of TAM biology. However, established tumors often sustain immune suppression through multiple redundant mechanisms, involving coordinated interactions between macrophages, tumor, and stromal and lymphocyte cells. This issue may account for the increasing necessity of using combination strategies 228. For this reason, future research should focus more on the rational combination of therapies, specifically TAM-targeted nanomedicine combined with immune checkpoint blockade, chemotherapy, radiotherapy, photothermal therapy, and other microenvironment-modulating strategies. Instead of simply amplifying treatment, the focus should be on using diverse and complementary therapeutic strategies that address the same target, TAMs 229.

Another problem focuses on targeting accuracy. Current methods typically depend on methods that passively facilitate phagocytic uptake, or on markers associated with CD206 and CD163. These markers are helpful and somewhat specific to tumor-associated macrophage populations, but not completely. Improved accuracy will require more than one control, including layers of selectivity associated with macrophage subsets, microenvironment-triggered activation, controlled release within the cell, and spatiotemporal changes in nanoparticle properties and behavior 230. In this case, with respect to these challenges, systems inspired by biology, nanocarriers coated with membranes, exosomes from macrophages, and vesicles that are engineered hold potential. However, these areas must incorporate more stringent approaches with regards to source control, reproducibility, and functionality 231.

Translation issues are also important. As discussed in the last section, antibodies, small-molecule inhibitors, and cell-based therapies are the most clinically advanced TAM-directed approaches. In addition, many sophisticated nanoplatforms remain at the preclinical level. Translation gaps reflect biological complexities and practical issues, such as limited penetration into tumors, off-target uptake by the mononuclear phagocyte system, problems associated with manufacturing scalability, batch-to-batch inconsistencies, and regulatory uncertainty. Therefore, developing TAM nanomedicine may require a more integrated translational framework considering target biology, delivery system design, pharmacokinetics, safety, and ease of manufacture 232.

A major priority for the field will be depleting, reprogramming, or utilizing macrophages as delivery carriers or immune effectors at defined macrophage manipulation windows. These goals cannot be substituted for one another and will depend on factors like choice of tumor, microenvironment, disease stage, as well as prior treatments. For instance, depletion may be necessary for reprogramming to an immune supportive state in the case of macrophages that are dominantly immunosuppressive and densely infiltrate the tumor. In instances where macrophages have inflammatory homing and tissue-penetrating abilities and support the precision of the delivery system, being engaged in delivery may be valuable. The development of myeloid-contextualizing approaches, such as local delivery of myeloid cells through implants after tumor resection, suggests next generation macrophage-associated nanomedicines may be required to move beyond generalized, system-wide delivery approaches to more defined, individualized delivery considering the treatment as well as the tumor 233.

TAM-targeted nanomedicine’s next focus must be on four priorities: selective identification of specific subsets of TAMs, development of multi-level systems with selective and penetrative targeting, rationally designing systems for combining treatments, and adopting an early stage of translatability in the design of technology 234. These goals will aid the field in moving beyond developing technologies for the sake of research and toward technologies that are truly therapeutically applicable.

## 6. Conclusion

TAM-targeted nanomedicine addresses the immunological, stromal, and resistance-associated roles of macrophages within the tumor microenvironment, making it a groundbreaking advancement in cancer treatment 235. The recent developments in techniques to block macrophage recruitment, tumor-supportive macrophage depletion, macrophage phenotype reprogramming to restore phagocytic function, and macrophage-derived delivery systems demonstrate TAMs’ pivotal role in tumor immunotherapy 236.

Significant advances in the field are anticipated to occur in parallel with the next decade of research efforts, as the development of approaches to tap into the therapeutic potential of nanomedicine is expected to target tumor-associated macrophages more effectively 237. For a tangible transformation of therapeutic tumor-associated macrophages nanomedicine from the research domain to the clinic, ongoing studies must prove their potential by overcoming the existing problems and achieving more lethal nanomedicines with cancer therapeutic efficacy.

## Figures and Tables

**Table 1:** Functional states and representative subsets of TAMs beyond the classical M1/M2 paradigm

TAM state / subset	Typical inducers	Representative markers	Major biological functions	Therapeutic relevance	Potential nanomedicine targeting implication	Representative references
M1-like / inflammatory TAMs	IFN-γ, LPS, Th1-associated inflammatory signals	MHC-II, TLR4, CD80, CD86, iNOS	Antigen presentation, pro-inflammatory cytokine secretion, ROS/NO production, and tumoricidal activity	Generally associated with immune-supportive antitumor activity; often used as a desired endpoint of TAM reprogramming rather than a rigid *in vivo* category.	Best used as a functional readout for reprogramming platforms, including NO/redox-responsive systems, TLR agonist nanocarriers, and intracellular signaling modulators.	35-38, 127-131, 143-147
M2-like / immunosuppressive TAMs	IL-4, IL-10, TGF-β, tumor-derived suppressive factors, hypoxia-associated cues	CD163, CD206, Arg-1	Immune suppression, tissue remodeling, angiogenesis support, tumor progression, and therapy resistance	Enriched in many tumor-promoting macrophage programs and commonly associated with poor prognosis; nevertheless, it should be considered a functional reference state rather than a single uniform population.	Frequently targeted through CD206-, CD163-, mannose-, or M2pep-related ligands, but selectivity is preferential rather than absolute and should be validated in each tumor context.	31-34, 38-45, 137-142
Angiogenic TAMs	Hypoxia, VEGF-rich TME, angiopoietin/TIE2-related cues	VEGF, PDGF, TGF-β, MMPs, TIE2 in relevant subsets	Promote neovascularization, vascular remodeling, extracellular matrix degradation, and abnormal vessel formation	Important drivers of tumor growth, abnormal perfusion, and drug-delivery barriers.	Provide rationale for anti-angiogenic TAM targeting, hypoxia-responsive systems, and platforms designed to reduce VEGF/MMP-associated signaling or reprogram angiogenic macrophage states.	52-60, 91-97
Immunosuppressive TAMs	IL-10, TGF-β, checkpoint-rich TME, chronic inflammatory suppressive signals	PD-L1, IL-10, IDO, arginase-1, CD39/CD73, galectin-9	Suppress CD8+ T-cell function, support Treg/MDSC recruitment, weaken antigen presentation, and contribute to immune escape	Major contributors to immune evasion, poor response to checkpoint blockade, and macrophage-centered suppression.	Relevant to immune-reprogramming nanomedicine, CD47/SIRPα blockade, CD40 activation, TLR agonist delivery, and checkpoint-combination strategies.	76-83, 164-178
Metastasis-associated TAMs	TGF-β, CCL18, EMT-associated inflammatory programs, tumor–stroma crosstalk	CCL18, TGF-β, matrix-remodeling phenotype, EMT-supportive mediators	Promote invasion, EMT, intravasation, pre-metastatic niche formation, metastatic dissemination, and colonization	Strongly linked to tumor spread, recurrence, and poor clinical outcome.	Support targeting of EMT-related, matrix-remodeling, exosome-mediated, and metastatic niche-supporting signals; may benefit from anti-metastatic combination nanotherapy.	68-75
Phagocytic-regulatory / efferocytic TAMs	Apoptotic cell burden, phosphatidylserine exposure, GAS6/Protein S, MerTK signaling	MerTK, phosphatidylserine-recognition machinery, CD276 in selected contexts	Efferocytosis, debris clearance, inflammation resolution, and tolerogenic immune regulation	In tumors, efferocytosis can reinforce immune tolerance and function as a macrophage-centered immune checkpoint.	Emerging target for efferocytosis-oriented nanomedicine, particularly MerTK-related blockade, BMS777607/UNC2025 delivery, or trigger-responsive local modulation.	152-163
Therapy-resistant / treatment-remodeling TAMs	Chemotherapy, radiotherapy, stress-induced cytokines, compensatory signaling, exosome-mediated crosstalk	IL-6/STAT3-associated phenotype, PI3K/Akt- and NF-κB-related survival programs, CSC-supportive signals	Promote chemoresistance, stromal adaptation, tumor cell survival, stem-like phenotypes, and relapse-associated remodeling	Important in treatment failure and recurrence; often requires combination with cytotoxic or immunotherapeutic strategies.	Justifies combining TAM-targeted nanomedicine with chemotherapy, radiotherapy, PI3Kγ/STAT inhibition, or resistance-modulating cargos.	84-90, 148-151, 195-203
Monocyte-derived TAM populations	CCL2/CCR2- and CSF-1/CSF-1R-dependent recruitment from circulation	Recruitment-associated macrophage phenotype; context-dependent markers after TME education	Rapidly replenish the TAM pool and maintain inflammatory or immunosuppressive myeloid niches	Particularly relevant in tumors with active inflammatory monocyte influx.	Most directly affected by recruitment-blocking strategies such as CSF-1R or CCR2/CCL2-directed nanomedicine; less effective against established tissue-resident macrophage pools.	26-30, 91-105
Tissue-resident macrophage-derived TAM populations	Local tissue niche signals, tumor-context adaptation, organ-specific stromal interactions	Organ- and niche-dependent markers; less uniformly defined than recruited TAMs	Provide persistent local support for tumor growth, stromal remodeling, and immune regulation	May be less affected by monocyte recruitment blockade alone and may require subset-aware reprogramming or local delivery approaches.	Highlights the need for spatial profiling, subset-specific ligands, and tumor-context-aware nanocarrier design rather than broad macrophage targeting.	22-27, 30, 40-45

**Table 2:** Major biological functions of TAMs in tumor progression and their therapeutic implications

Biological process	Major TAM-derived mediators / pathways	Consequence for tumor progression	Corresponding therapeutic rationale	Related TAM-targeted strategy class	Representative references
Angiogenesis	VEGF, PDGF, TGF-β, MMPs, hypoxia/HIF signaling, TIE2-associated TAM programs	Promotes neovascularization, vascular remodeling, extracellular matrix degradation, abnormal perfusion, and impaired drug delivery.	Suppress macrophage-driven pro-angiogenic signaling, limit hypoxia-associated TAM activation, and normalize the vascular microenvironment.	Recruitment blockade; TAM depletion; macrophage reprogramming; hypoxia- or pH-responsive nanomedicine.	52-60, 91-97, 127-131
Lymphangiogenesis	VEGF-C, VEGF-D, VEGFR-3, lymphatic endothelial activation, hypoxia- and prostanoid-associated lymphangiogenic signaling	Enhances lymphatic vessel formation, lymphatic permeability, lymph node metastasis, and regional dissemination.	Disrupt TAM-driven lymphatic signaling and reduce macrophage-mediated support for lymphatic metastatic spread.	Macrophage reprogramming; anti-metastatic pathway-directed delivery; TAM depletion in lymphangiogenic niches.	61-67
Metastasis / EMT	TGF-β, CCL18, TNF-α, IL-10-associated inflammatory signaling, MMPs, exosome-mediated intercellular communication	Promotes tumor cell invasion, EMT, intravasation, pre-metastatic niche formation, and distant colonization.	Inhibit TAM-mediated EMT induction, matrix remodeling, and metastatic niche support while interrupting macrophage-tumor crosstalk.	Macrophage reprogramming; anti-metastatic combination nanotherapy; efferocytosis-related regulation; biomimetic delivery systems.	68-75, 152-163, 186-203
Immune evasion	PD-L1, IL-10, TGF-β, IDO, arginase-1, CD39/CD73, galectin-9/Tim-3, CD47/SIRPα-related phagocytic suppression	Suppresses cytotoxic T-cell activity, promotes Treg/MDSC accumulation, reduces antigen presentation, and protects tumor cells from macrophage phagocytosis.	Restore innate and adaptive antitumor immunity by reversing macrophage-mediated immune suppression and reactivating phagocytic or antigen-presenting functions.	CD47/SIRPα blockade; CD40-mediated immune activation; TLR agonist-based repolarization; CAR-M; immune-combination nanomedicine.	76-83, 143-147, 164-185
Chemotherapy resistance	IL-6/STAT3, PI3K/Akt, NF-κB, CSC-supportive signaling, abnormal angiogenesis, exosome-mediated resistance transfer	Reduces drug sensitivity, supports tumor cell survival and stemness, impairs intratumoral drug delivery, and contributes to recurrence.	Combine TAM modulation with cytotoxic therapy, improve tumor drug penetration, and disrupt resistance-supportive macrophage signaling and exosome communication.	PI3Kγ/STAT-targeted reprogramming; phagocytosis-restoring approaches; biomimetic and exosome-based delivery platforms; multimodal combination nanomedicine.	84-90, 148-151, 195-203

**Table 3:** Concise summary of representative TAM-targeted nanomedicine strategies

Strategy class	Representative targets / platforms	Core nanocarrier design logic	Main TAM-related effect	Key strength and key limitation	Refs.
Blocking macrophage recruitment	CSF-1/CSF-1R: BLZ945 micelles, BLZ-945SCNs/Pt, CSF-1R siRNA NPsCCL2/CCR2: CCR2 siRNA NPs, BisCCL2/5i mRNA LNPs, KLAK-MCP-1 micelles	pH-responsive release; chemokine/receptor-guided targeting; RNA or small-molecule delivery; ligand-assisted macrophage enrichment	Limits inflammatory monocyte recruitment, TAM replenishment and CSF-1R-dependent survival; remodels macrophage-rich TME	Strength: strong biological/clinical rationale; combination-friendly. Limitation: chemokine redundancy, adaptive resistance, incomplete effect on tissue-resident macrophages.	91-105, 206-210
Depleting tumor-promoting TAMs	Liposomal clodronate; zoledronic acid liposomes/nanocarriers; legumain-responsive ATpep-NPs, PPP and s-Tpep-NPs	Phagocytic liposomal uptake; enzyme-cleavable linkers; PEG shedding; activation in legumain-rich/hypoxic tumor regions	Reduces tumor-promoting macrophage burden; may improve co-delivered drug retention or tumor-restricted activation	Strength: direct burden reduction; legumain systems improve spatial control. Limitation: nonselective phagocyte depletion, TAM heterogeneity and trigger variability.	106-120
Reprogramming TAM polarization and function	CPHT; DMON-SNO; miR-99b delivery; GO-PEG-PEI/CpG; HA-Man NC; DOX@MAN-BSANP; CDNP-R848; R848 nanoemulsion; SVMAV	Redox/NO-responsive release; size-dependent TLR engagement; CD206/MR-guided uptake; TLR agonist loading; local immune activation	Redirects suppressive/M2-like TAMs toward immune-supportive states and enhances innate-adaptive immunity	Strength: preserves macrophages while changing function; broad cargo compatibility. Limitation: incomplete/unstable repolarization and inflammatory toxicity risk.	127-147
Intracellular signaling and efferocytosis-oriented reprogramming	PI3Kγ/STAT3: mannose iron oxide NPs, STAT3/HIF-1α siRNA LNPs, CD163-targeted liposomesEfferocytosis/MerTK: DOX+BMS777607 silica NPs, UNC2025 NPs, siMerTK LNPs	Macrophage-enriched ligands; pH-sensitive intracellular delivery; synchronized chemotherapy/efferocytosis blockade; MerTK-oriented delivery	Suppresses immunosuppressive signaling or tumor-supportive apoptotic-cell clearance, promoting antitumor activation	Strength: mechanistically specific and linked to clinical pathways. Limitation: pathway pleiotropy, efferocytosis homeostasis concerns and early nano evidence.	148-163, 212
Restoring phagocytic and immune-activating functions	CD47/SIRPα: M1 exosome-antibody systems, ROS albumin NPs, CD47 blockade + calreticulin NPsCD40: CD40 mRNA LNPsCAR-M: HER2 CAR-M, HER2/CD47 CAR-M, glioma CAR-M, MPEI/pCAR-IFN-γ	pH/ROS-triggered antibody release; “don’t eat me” blockade plus “eat me” signals; mRNA LNPs; *ex vivo* or *in situ* macrophage programming	Restores tumor-cell engulfment, antigen presentation and innate-adaptive immune crosstalk	Strength: links phagocytosis with T-cell immunity; CD47 and CAR-M are clinically advanced concepts. Limitation: off-tumor toxicity, immune activation and manufacturing complexity.	164-185, 211, 213-215
Biomimetic and macrophage-derived delivery platforms	Macrophage membrane-coated NPs: cRGD membrane nanovesicles, M@BS-QE-NP, NMm-PLGA/RAPAEngineered exosomes/EVs: DTX-M1-Exo, M1 EV/siCX3CR1, E-cLip-DTX/si, CAR-EDC	Membrane camouflage; hybrid membrane design; donor polarization control; exosome/liposome hybridization; trigger-responsive linkers	Improves inflammatory homing, payload protection, biodistribution and spatial precision; may also provide immune modulation	Strength: modular framework for delivery, depletion, reprogramming or phagocytosis restoration. Limitation: source variability, vesicle heterogeneity, scale-up and GMP/QC challenges.	186-203, 224

**Table 4:** Current clinical candidates, translational barriers, and future opportunities in TAM-targeted therapy

Target / strategy	Representative agent	Modality	Clinical stage	Tumor type	Major finding	Main barrier	Future opportunity	Representative references
CSF1/CSF1R inhibition	Pexidartinib	Small molecule	Approved	Tenosynovial giant cell tumor (TGCT)	Provides clinical proof that macrophage-directed CSF1R inhibition can be actionable.	Limited applicability beyond selected macrophage-dependent indications.	Biomarker-guided extension to macrophage-rich tumors and rational combinations.	206
CSF1R blockade	Emactuzumab	Antibody	Phase I	Advanced solid tumors	Demonstrated pharmacodynamic depletion of immunosuppressive M2-like macrophages.	Limited single-agent efficacy in many solid tumors.	Combination with chemotherapy, immune checkpoint blockade, or patient-selection biomarkers.	207
CSF1R-targeted combination	LY3022855 with durvalumab or tremelimumab	Antibody / pathway inhibitor combination	Phase Ia/Ib	Advanced solid tumors	Confirms feasibility of CSF1R-based combination therapy, but clinical activity remained modest.	Toxicity, dose optimization, and modest antitumor activity.	Improved patient stratification and regimen design.	208
CCR2-mediated recruitment blockade	PF-04136309	Small molecule	Phase Ib	Pancreatic ductal adenocarcinoma	Provides proof-of-concept for inflammatory monocyte/TAM recruitment control in pancreatic cancer combinations.	Chemokine redundancy, regimen dependence, and limited durability.	Use in monocyte-inflamed tumors with rational chemotherapy or immunotherapy combinations.	103, 209, 210
CD47/SIRPα checkpoint blockade	Magrolimab plus cetuximab	Antibody combination	Phase Ib/II	Colorectal cancer and other solid tumors	Supports macrophage phagocytosis-restoring strategy with tolerability and signs of antitumor activity.	On-target/off-tumor toxicity due to CD47 expression on normal cells; safety-window concerns.	Tumor-restricted delivery, biomarker-guided combinations, and nanocarrier-enabled local release.	164-166, 211
PI3Kγ-directed macrophage reprogramming	Eganelisib (IPI-549)	Small molecule	Phase I/Ib	Advanced solid tumors	Clinically validates myeloid-state reprogramming as a feasible strategy; activity observed as monotherapy and with nivolumab.	Limited single-agent efficacy; pathway pleiotropy and context dependence.	Combination with checkpoint inhibitors in myeloid-dominant tumors; biomarker-defined patient selection.	150, 212
CAR-macrophage therapy	CT-0508	Cell therapy	Phase I	HER2-overexpressing solid tumors	Demonstrates feasibility, tolerability, tumor trafficking, and TME remodeling in early clinical testing.	Manufacturing complexity, persistence, biodistribution, scalability, and antigen heterogeneity.	Combination with checkpoint blockade and optimized macrophage engineering or local/*in situ* programming.	213-215
Multifunctional TAM-targeted nanoplatforms and biomimetic systems	Representative preclinical nanocarriers, membrane-coated particles, and engineered exosomes	Nanomedicine / biomimetic delivery	Mostly preclinical	Various solid tumor models	Strong preclinical rationale and multifunctional design, but no comparable clinical dataset to pathway-directed drugs or cell therapies.	Complex composition, batch variability, biodistribution uncertainty, MPS sequestration, GMP scale-up, and QC requirements.	Early integration of manufacturability, standardized characterization, local delivery formats, and clinical benchmarking.	204, 216-225

## Abbreviations

APC: antigen-presenting cell
Arg-1: arginase-1
BBB: blood-brain barrier
Bcl-2: B-cell lymphoma 2
CAR-M: chimeric antigen receptor macrophage
CAR-T: chimeric antigen receptor T cell
CCL17: C-C motif chemokine ligand 17
CCL18: C-C motif chemokine ligand 18
CCL2: C-C motif chemokine ligand 2
CCL22: C-C motif chemokine ligand 22
CCL5: C-C motif chemokine ligand 5
CCR2: C-C chemokine receptor 2
cGAS-STING: cyclic GMP-AMP synthase-stimulator of interferon genes
CSC: cancer stem cell
CSF-1: colony-stimulating factor 1
CSF-1R: colony-stimulating factor 1 receptor
CT-0508: anti-HER2 chimeric antigen receptor macrophage product
CX3CR1: C-X3-C motif chemokine receptor 1
DC: dendritic cell
DOX: doxorubicin
DTX: docetaxel
ECM: extracellular matrix
EMT: epithelial-mesenchymal transition
EV: extracellular vesicle
GAS6: growth arrest-specific 6
GMP: good manufacturing practice
GO: graphene oxide
GSH: glutathione
HA: hyaluronic acid
HER2: human epidermal growth factor receptor 2
HIF: hypoxia-inducible factor
HIF-1α: hypoxia-inducible factor 1α
ICB: immune checkpoint blockade
IDO: indoleamine 2,3-dioxygenase
IFN-γ: interferon-γ
IGF-1: insulin-like growth factor 1
IGF-1R: insulin-like growth factor 1 receptor
IL: interleukin
iNOS: inducible nitric oxide synthase
LNP: lipid nanoparticle
LPS: lipopolysaccharide
MDSC: myeloid-derived suppressor cell
MerTK: MER proto-oncogene tyrosine kinase
MHC-II: major histocompatibility complex class II
MMP: matrix metalloproteinase
MPEI: mannose-conjugated polyethyleneimine
MPS: mononuclear phagocyte system
MR: mannose receptor
mRNA: messenger RNA
NF-κB: nuclear factor-κB
NO: nitric oxide
NP: nanoparticle
PD-1: programmed cell death protein 1
PD-L1: programmed death-ligand 1
PDAC: pancreatic ductal adenocarcinoma
PDGF: platelet-derived growth factor
PEG: polyethylene glycol
PEI: polyethyleneimine
PI3K: phosphoinositide 3-kinase
PI3Kγ: phosphoinositide 3-kinase γ
PLGA: poly(lactic-co-glycolic acid)
PS: phosphatidylserine
QC: quality control
R848: resiquimod
ROS: reactive oxygen species
siRNA: small interfering RNA
SIRPα: signal regulatory protein α
STAT3: signal transducer and activator of transcription 3
TAM: tumor-associated macrophage
TGCT: tenosynovial giant cell tumor
TGF-β: transforming growth factor-β
TIE2: tyrosine kinase with immunoglobulin-like and EGF-like domains 2
TIL: tumor-infiltrating lymphocyte
Tim-3: T-cell immunoglobulin and mucin-domain containing-3
TLR: Toll-like receptor
TME: tumor microenvironment
TNBC: triple-negative breast cancer
TNF-α: tumor necrosis factor-α
Treg: regulatory T cell
VEGF: vascular endothelial growth factor
VEGF-A: vascular endothelial growth factor A
VEGF-C: vascular endothelial growth factor C
VEGF-D: vascular endothelial growth factor D
VEGFR-3: vascular endothelial growth factor receptor 3
ZOL: zoledronic acid
